# Immunosenescence, Physical Exercise, and their Implications in Tumor Immunity and Immunotherapy

**DOI:** 10.7150/ijbs.100948

**Published:** 2025-01-06

**Authors:** Xin Yu, Wei Pei, Bei Li, Shengrong Sun, Wenge Li, Qi Wu

**Affiliations:** 1 Department of Breast and Thyroid Surgery, Renmin Hospital of Wuhan University, Wuhan, Hubei, P. R. China.; 2 Tongji University Cancer Center, Shanghai Tenth People's Hospital, School of Medicine, Tongji University, Shanghai, P. R. China.; 3 Department of Pathology, Renmin Hospital of Wuhan University, Wuhan, Hubei, P. R. China.; 4 Department of Oncology, Shanghai GoBroad Cancer Hospital, Shanghai, P. R. China.

**Keywords:** Exercise, Immunosenescence, Tumor Immunity, Immunotherapy

## Abstract

Aging is associated with a decline in immune function, termed immunosenescence, which compromises host defences and increases susceptibility to infections and cancer. Physical exercise is widely recognized for its myriad health benefits, including the potential to modulate the immune system. This review explores the bidirectional relationship between immunosenescence and physical exercise, focusing on their interplay in shaping antitumor immunity. We summarize the impact of aging on innate and adaptive immune cells, highlighting alterations that contribute to immunosenescence and cancer development. We further delineate the effects of exercise on immune cell function, demonstrating its potential to mitigate immunosenescence and enhance antitumor responses. We also discuss the implications of immunosenescence for the efficacy of immunotherapies, such as immune checkpoint inhibitors and adoptive T cell therapy, and explore the potential benefits of combining exercise with these interventions. Collectively, this review underscores the importance of understanding the complex relationship between immunosenescence, physical exercise, and antitumor immunity, paving the way for the development of innovative strategies to improve cancer outcomes in the aging population.

## Introduction

The intricate relationship between the aging process and the concurrent decline in immune system efficacy has been extensively studied^1^-^3^. With the global population aging, it is essential to comprehend the mechanisms behind the age-related decline in immune function, known as immunosenescence. This understanding is crucial for tackling the rising incidence of age-associated diseases, including cancer. And the dynamic relationship among the immune response, physical activity, and the shifting paradigm of antitumor immunity is a major research hotspot at present^4^,^5^.

Immunosenescence is characterized by alterations in cellular composition and immune function, which result in attenuated reactions to pathogens and an increased susceptibility to various chronic conditions^1^,^6^. One such disease that prominently manifests with age is cancer, in which the compromised immune system contributes to the development and progression of malignant tumors^7^. Given the intricate relationship between aging and cancer, developing strategies for delaying immunosenescence and enhancing antitumor immunity is a prime focus of ongoing research.

Physical exercise, widely acknowledged for its multifaceted health benefits, has recently gained attention for its potential to modulate the immune system^8^. Numerous studies have demonstrated that regular exercise can positively affect immune function, attenuating the detrimental effects of immunosenescence^9^,^10^. The mechanistic basis of this positive effect involves the modulation of inflammatory mediators, enhancement of immune cell activity, and formation of an immune environment conducive to effective antitumor responses^11^,^12^.

In recent years, the field of tumor immunology has witnessed remarkable progress with the advent of immunotherapy^13^,^14^. Various types of cancer immunotherapies, such as immune checkpoint inhibitors (ICIs), adoptive cell transfer, cytokine treatments, oncolytic virus therapy and cancer vaccines, have emerged and demonstrated potential in clinical settings. Immunotherapy harnesses the body's innate immune system to selectively identify and eradicate cancer cells, representing a transformative strategy in cancer treatment^13^-^15^. Understanding the complex relationship among immunosenescence, physical exercise, and antitumor immunity is essential for optimizing the efficacy of immunotherapy, particularly in the context of an aging population.

This review provides a comprehensive summary of immunosenescence, the influence of physical activity upon immune system functionality, and the roles of these factors in shaping antitumor immunity, aiming to facilitate the development of innovative and personalized strategies for treating age-related diseases and improving the outcomes of immunotherapy.

## Aging and the immune system

**Figure 1:**
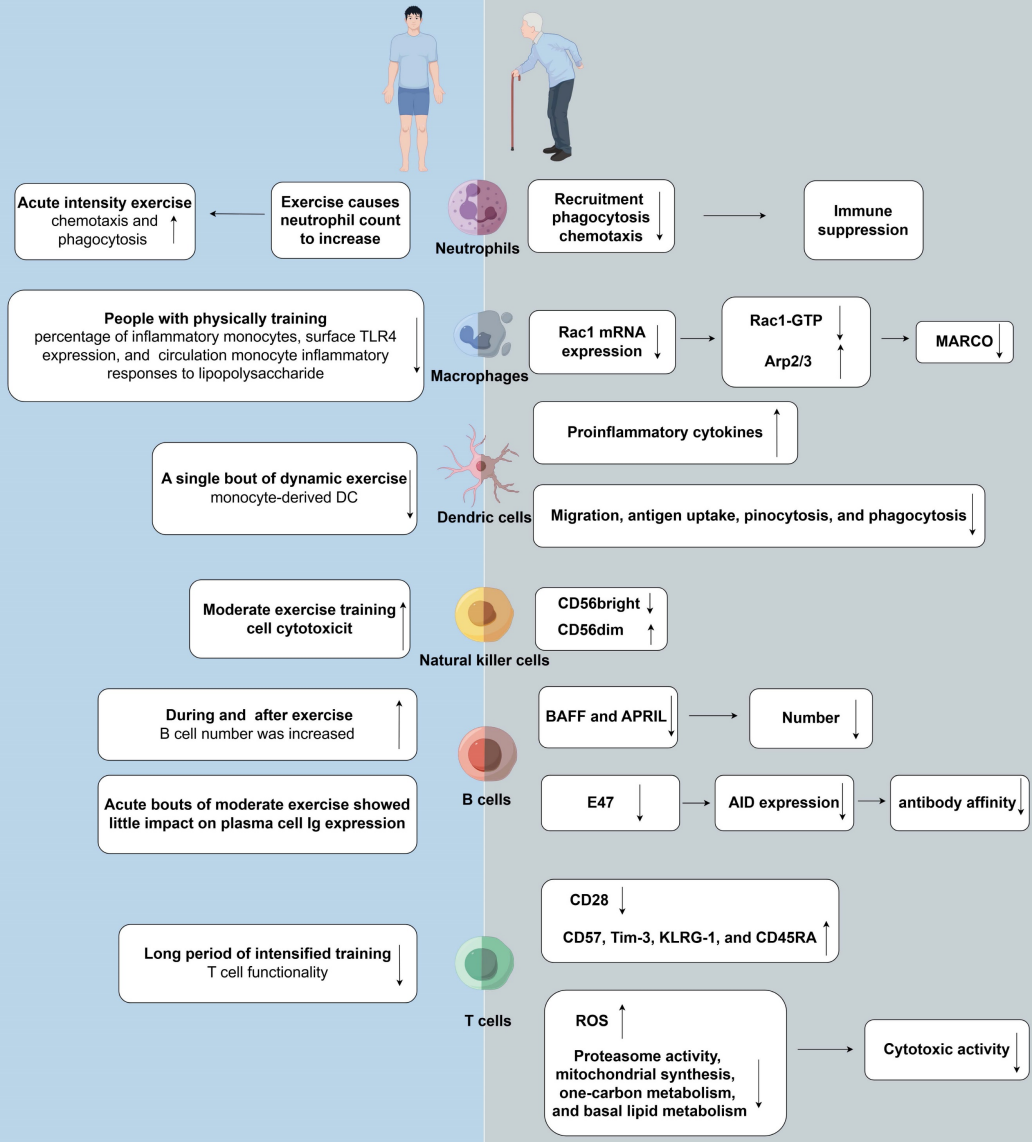
** Effects of aging and exercise on tumor immunity.** (right) Immunosenescence weakens the immune system's capability to effectively respond to pathogens and vaccines. Neutrophil functional decline with aging includes reduced chemotaxis, phagocytosis, and NET production. Macrophages exhibit diminished phagocytic activity and antigen presentation. Aging impairs dendritic cell function, affecting antigen-presenting efficiency, cytokine production, and immune coordination. NK cell cytotoxicity decreases despite stable or slightly increased numbers. B cells decline in number and function with age, reducing antibody production. Immunosenescence involves stable T cell counts but reduced naïve T cells and increased senescent CD28-negative memory T cells, leading to compromised immune function and susceptibility to diseases. (left) Physical exercise has the potential to enhance immune function by counteracting the detrimental effects of aging. Engaging in a single session of physical exercise alters circulating neutrophil quantity and distribution, with high-intensity resistance and endurance exercises causing temporary increases that return to baseline within hours. Regular physical activity reduces baseline CD14lowCD16+ inflammatory monocytes, linked to decreased TLR4 expression and subdued inflammatory responses to LPS. Dynamic exercise boosts monocyte-derived dendritic cell production, impacting immune responses variably. NK cells are pivotal in exercise immunology due to their modulation by physical activity, affecting functionality and abundance post-exercise. Physical exercise affects humoral immunity, influencing systemic Ig levels and salivary sIgA, which bolster mucosal defenses. T cell proliferation is generally suppressed by physical exertion, notably impacting Th1 cells in athletes, with CD4+ and CD8+ T cell counts fluctuating post-exercise. Rac1: ras-related C3 botulinum toxin substrate 1; Arp: actin-related proteins; MARCO: macrophage receptor with collagenous structure; BAFF:B-cell-activating factor; APRIL: proliferation-inducing ligand.

Immunosenescence, defined by the reduction in immune functionality as one ages, involves a multifaceted set of biological processes. This includes significant organ remodeling and diverse cellular regulatory mechanisms^2^. Senescent cells secrete a variety of factors known as the senescence-associated secretory phenotype (SASP), which not only promote chronic inflammation but also induce senescence in neighboring healthy cells. Simultaneously, this persistent inflammatory state accelerates immune cell senescence, impairing immune function and preventing the clearance of both senescent cells and pro-inflammatory mediators. This creates a vicious cycle of inflammation and cellular senescence. Prolonged inflammation in organs such as the lungs, liver, and bone marrow can hinder effective clearance, contributing to organ damage and the development of age-related pathologies^16^. Immunosenescence attenuates the ability of the immune system to induce effective responses against pathogens and vaccinations. Although the comprehensive landscape of underlying changes remains elusive, some notable alterations include dysfunction of hematopoietic stem cells, an altered naive-to-memory cell ratio within T and B cell populations, inflammaging, accumulation of senescent cells, weak responses to novel antigens, and altered stress responses^17^,^18^ (Fig. 1).

### Innate immune cells

#### Neutrophils

Neutrophils serve as the first line of defense against infections and are the most abundant circulating cells in human blood, rapidly recruited to injury sites^19^. Traditionally, it was believed that their inability to proliferate post-maturation leads to rapid exhaustion and a short half-life. This limited understanding suggested neutrophils had a narrow functional role in immune defense, unlike other myeloid cells. Recently, however, this view has been challenged. Studies indicate that neutrophils may survive in circulation for up to 5.4 days, and their lifespan can extend following activation. Additionally, neutrophils can undergo reverse transmigration from injury sites back into circulation, although the reasons for this phenomenon remain unclear. Evidence suggests that these recirculating neutrophils may enhance T cell proliferation and responses by migrating to lymphoid organs^20^.

Despite the preservation of neutrophil count in aged individuals, the functional abilities of these cells—such as chemotaxis, phagocytosis, and the production of NETs—deteriorate due to the aging process^21^,^22^. Studies investigating changes in the quantity and function of neutrophils in geriatric animal models are relatively limited. In a study by Nacionales *et al.*, transcriptomic analysis showed that geriatric mice with sepsis exhibited weaker neutrophil mobilization, phagocytosis, and chemotaxis than their younger counterparts^23^. Additionally, a decreased accuracy in chemotaxis, along with a compromised ability to clear apoptotic cells (efferocytosis) and generate immune responses, has been associated with a prolonged state of immunosuppression^24^. However, further investigation is warranted to elucidate the alterations in the count, phenotype, and function of neutrophils among elderly patients.

#### Monocytes/Macrophages

Tissue-resident macrophages originate from circulating monocytes and are tissue-specific. They defend against pathogens via phagocytosis and cytokine release, activating innate immunity^25^. They also present antigens to boost adaptive immunity^26^.

With advancing age, the overall count of macrophages within the body remains relatively stable; however, their function is remarkably altered. Macrophages can be categorized into two subsets as follows: M1, characterized by pro-inflammatory and anti-tumorigenic properties, and M2, characterized by anti-inflammatory and pro-tumorigenic properties^27^. The debate surrounding the occurrence of macrophages in a state of senescence within living organisms remains an area of contentious discussion and ongoing investigation in the field of biology. In the geriatric population, M1 macrophages are predominant in healthy hepatic and adipose tissues, whereas M2 macrophages, characterized by their immunosuppressive activity, are predominant in bone marrow, lymphoid, spleen, muscular, and pulmonary tissues^28^-^31^. Macrophages exhibiting M2-like characteristics have been shown to promote angiogenesis in geriatric mice with injury. This observation implies a correlation between senescent macrophages and the development of numerous age-associated disorders, with a particular emphasis on neoplastic conditions^32^.

Aging is associated with a marked decrease in macrophage phagocytic activity and a concomitant reduction in surface expression of Toll-like receptors (TLRs)^26^. The reduction in TLR expression levels has been associated with a concurrent rise in the proportion of regulatory T cells (Tregs)^33^. Evidences showed that macrophages from aged mice demonstrate reduced reactivity to TLR-1, TLR-2, and TLR-4. This attenuated response may be attributed to the decreased synthesis of important pro-inflammatory cytokines, such as interleukin (IL)-6, IL-1β, and tumor necrosis factor-alpha (TNF-α), owing to weakened activation of the NF-κB, p38, and JNK pathways^34^,^35^. Li *et al.* revealed that the phagocytic activity of macrophages is compromised, at least in part, owing to a reduction in the expression of Rac1 mRNA. This downregulation decreases the expression of Rac1-GTP and suppresses the activation of the Arp2/3 complex, resulting in decreased polymerization of F-actin, impaired formation of filopodia, and reduced surface expression of MARCO (a receptor crucial for the efficient engulfment of pathogens through phagocytosis)^36^.

Furthermore, the decline in the antigen-presenting capacity of macrophages to CD4 T lymphocytes has been linked to aging. This decline predominantly stems from the reduced expression of major histocompatibility complex class II (MHC-II) molecules, particularly the human leukocyte antigen (HLA)-DR isotype^37^. Efferocytosis, which refers to the phagocytic removal of apoptotic cells, has been shown to decline with age. The age-dependent decline in efferocytosis can impair the role of macrophages in the resolution of infections and modulation of inflammatory responses, consequently increasing the risk of tissue damage^38^. Accumulation of senescent tissue-resident macrophages and neutrophils has been shown to promote chronic, low-grade inflammation. This persistent inflammatory state contributes to immunosuppression mediated by macrophages, eventually driving the initiation of various diseases, including cancer^39^. In aged mice harboring neoplasms, the proportion of myeloid-derived suppressor cells (MDSCs) within the bone marrow, peripheral blood, and spleen is notably high. The increased abundance of MDSCs constitutes a critical barrier, as these cells are involved in mediating immunosuppression. Consequently, persistent immunosuppression not only impedes the removal of senescent cells but also prevents the destruction of tumor cells while disrupting tissue protein homeostasis and energy metabolism^40^. Cells characterized by the senescence-associated secretory phenotype (SASP) release various chemotactic and inflammatory mediators. Mediators such as chemokines and cytokines play a pivotal role in orchestrating the recruitment of MDSCs to the tumor microenvironment (TME). This recruitment process not only fosters immune evasion but also enhances metastatic potential, thus underscoring their significance in tumor progression and metastasis facilitation^40^. He *et al.* showed that MDSCs can modulate T-cell responses during the embryonic and fetal phases of development in mice and humans. Additionally, accumulation of MDSCs can indicate an underlying pathological condition or can be a physiological response to pregnancy^41^. Excessive proliferation of MDSCs has been shown to promote immunosenescence. An increased proportion of MDSCs can lead to detrimental secondary effects on tissue integrity. This outcome is primarily attributed to the cellular production of cytokines, including IL-10 and transforming growth factor beta (TGF-β), which influence tissue integrity and regulation of immune responses^42^.

#### Dendritic Cells

Dendritic cells (DCs) are essential antigen-presenting cells (APCs) that initiate adaptive immune responses by internalizing antigens and presenting them to T cells^43^. Activation occurs via pattern recognition receptors (PRRs) upon encountering pathogens, leading to increased expression of MHC class I and II molecules and co-stimulatory molecules like CD40, CD80, and CD86. This process also triggers the secretion of proinflammatory cytokines such as IFN-γ, TNF-α, IL-6, and IL-12, enhancing T-cell priming^43^,^44^. DCs are categorized into myeloid DCs (mDCs), which effectively present antigens and secrete cytokines, and plasmacytoid DCs (pDCs), which are key producers of type I IFN^43^,^45^.

Although a reduction in the number of DCs is commonly associated with aging, recent studies have suggested that, in some cases, the DC count is relatively stable or moderately increased in elderly individuals^46^. On the contrary, the functionality of DCs is substantially altered with advancing age^46^. Evidences showed that DCs obtained from elderly individuals had an enhanced proinflammatory profile characterized by elevated baseline synthesis of cytokines, including IL-6 and TNF-α. This inflammatory condition was potentially associated with SASP, a hallmark of immunosenescence^47^-^49^. Li *et al.* highlighted in their study that there exists diminished expression of costimulatory molecules among CD8α DCs isolated from aged mice. These molecules play a crucial role in facilitating interactions with MHC class II and CD40, pivotal for effective T-cell priming. This observed downregulation potentially hinders the capacity of DCs to elicit robust T-cell priming responses^50^. DCs from elderly individuals often exhibit a reduction in their quantity, HLA-DR expression, and functional efficiency, particularly in terms of migration, antigen presentation, pinocytosis, and phagocytosis^51^. Despite the important role of DCs in orchestrating immune responses, immunomodulatory therapies specifically targeting DCs are lacking. The cytokine FLT3L, known for its role in DC proliferation, has been shown to ameliorate immunosuppression triggered by sepsis^52^,^53^. In addition, IL-15 has been shown to exert efficient immunostimulatory and proliferation-inducing effects on DCs^54^.

MDSCs, which are associated with immunosenescence, can negatively affect the functionality of DCs^55^. Studies have shown that stimulation of MDSCs with lipopolysaccharides (LPSs) and IFN-γ can disrupt DC maturation DCs in mouse bone marrow cultures^56^. Additionally, DCs co-cultured with human MDSCs exhibit diminished antigen-presenting capabilities and suppressed cytokine synthesis^57^. TGF-β1 has been identified as an effective inhibitor of the maturation and functional activity of DCs in both humans and murine species^58^. The role of TGF-β1 in the mouse epidermis is pivotal for the development and maintenance of Langerhans cells, exhibiting variable effects across different subsets of DCs^59^. Therefore, MDSCs may inhibit the function of DCs, potentially resulting in the attenuation of T- and B-cell responses in inflammation and cancer.

#### Natural killer cells

Natural killer (NK) cells, a subset of lymphocytes, significantly bolster the adaptive immune system. Their pivotal function lies in stimulating both innate and adaptive immune reactions, particularly targeting cancer cells^60^. They can not only deliver a rapid, non-specific attack on cells hosting pathogens^61^ but also relocate and facilitate prompt reactions by enhancing the function of myeloid lineage cells, notably macrophages, through the secretion of IFN-γ^60^,^61^. In human peripheral blood, NK cells are categorized into CD56bright and CD56dim subsets, distinguished by their differential expression of the homophilic adhesion molecule CD56. The former subtype is associated with cytokine production, whereas the latter is associated with cytotoxicity^62^.

The number of NK cells typically remains unaltered or exhibits a slight increase in elderly individuals^63^. This moderate increase can be attributed to the higher proportion of the mature CD56dim subtype and the lower proportion of the immature CD56bright subtype^64^. The IFN-γ production in NK cells either remains unaltered or potentially escalates as age progresses, however, their cytotoxic capacity is frequently reduced, possibly owing to the decreased secretion of perforin into the immunological synapse^65^,^66^. NK cells possess activating receptors such as NKp30, NKp46, and DNAM-1, which are important for the identification and elimination of different tumor cell types, including hematological cancer^67^, melanoma^68^, and ovarian cancer^69^ cells. Researchers have hypothesized that alterations in the expression profiles of these receptors among older individuals could impact the capacity of NK cells in surveilling and eradicating malignant cells^70^,^71^. For older individuals who have been diagnosed with acute myeloid leukemia (AML), the NK cell population shows unique changes. This is manifested by a decrease in the generation of immature CD56 bright cells and a simultaneous increase in the generation of highly differentiated CD56 dim cells. These changes are suggestive of disease progression and function as prognostic indicators^72^-^74^. A clinical study that involved the infusion of killer cell immunoglobulin-like receptor (KIR) ligand-mismatched NK cells into elderly patients with AML after immunosuppressive chemotherapy validated the safety and applicability of NK cell transplantation within this specific patient group^75^.

### Adaptive Immune Cells

#### B cells

B cells, derived from the bone marrow, are essential components of the adaptive immune system and crucial in coordinating both innate and adaptive immune responses^76^,^77^. They can identify a wide spectrum of antigens, encompassing proteins, lipids, polysaccharides, nucleic acids, and diverse chemicals. The precision of B cells in antigen recognition is substantially increased through their interaction with B-cell receptors (BCRs), and this process is further enhanced by the synergistic action of helper T cells^76^,^77^.

Upon receiving co-stimulatory signals, immature B lymphocytes undergo activation, followed by cell division and differentiation into plasma cells. These differentiated cells have the unique ability to synthesize highly specific antibodies. After an infection has been resolved, some plasma cells form memory B cells, which trigger a rapid and enhanced response to the same pathogen upon future encounters^78^. Furthermore, B cells, by producing antigen-specific immunoglobulins (Igs) and undergoing class switching, play a pivotal role in humoral immunity. They can also act as APCs, activating T cells by expressing MHC class II molecules^79^,^80^.

Advancing age is accompanied by a concurrent decline in both the number of B cells and the functional capacity of hematopoietic stem cells within the bone marrow^81^,^82^. Additionally, the circulatory levels of two vital growth factors, namely, a proliferation-inducing ligand (APRIL) and B-cell-activating factor (BAFF), are remarkably reduced in elderly individuals. The reduced expression of these factors in the plasma of older individuals correlates with a decline in B cell count, which is essential for maintaining mature B cells in peripheral blood^83^.

As age progresses, there is a decline in the functional capacity of B cells, resulting in diminished humoral immune responses and decreased antibody efficacy. The diminished functionality of cells leads to reduced antibody titers and diminished binding affinity. This outcome arises from the intricate interaction between intrinsic and extrinsic factors^84^. An important intrinsic factor is the decreased ability of B cells to form germinal centers, which affects somatic hypermutation and affinity maturation. This state is worsened by a decline in the expression of activation-induced cytidine deaminase (AID) due to the downregulation of the transcription factor E47. As a result, class switch recombination and affinity maturation are prevented^85^. The decline is contributed to by alterations in BCR signaling and reduced expression levels of costimulatory molecules, such as CD86^82^. Furthermore, the transformation of a significant portion of naive B cells into memory B cells poses limitations on the production of high-affinity antibodies against new antigens^86^.

The age-related decline in CD4+ T helper cell function, along with other extrinsic factors, plays a pivotal role in the regulation of B-cell function. While CD4+ T helper cells are crucial for germinal center formation, somatic hypermutation initiation, and high-affinity antibody synthesis in B cells, their effectiveness diminishes with age^87^,^88^. Altogether, the intrinsic and extrinsic changes in B-cell dynamics synergistically attenuate the humoral immune response in elderly individuals. Despite the integral role of B cells in coordinating immune responses, studies developing therapeutic strategies for modulating their activity are limited. A relevant study showed that androstenediol, a derivative of dehydroepiandrosterone (DHEA), enhanced B-cell responses in a murine model of aging^89^. This finding highlights the potential therapeutic value of DHEA-derived metabolites in regulating B-cell function^89^.

#### T cells

During immunosenescence, a notable inconsistency is observed between the unaltered total T cell count and the remarkable heterogeneity among T cell subpopulations throughout life. A hallmark characteristic of this diversity lies in the reduced fraction of naïve T cells, concomitant with an escalation in the fraction of highly differentiated CD28-negative memory T cells, often denoted as senescent T cells^90^. Upon encountering antigens, T cells experience proliferation and subsequently differentiate into a diverse array of effector T cell clones, each characterized by distinct functional attributes^91^. A specific subset of clones among these exhibits sustained specificity towards antigens, impacting both humoral and cell-mediated immune responses. The classification of T cells based on phenotype relies on the expression of distinct cell surface markers, primarily CD molecules, and the cytokines they secrete. T helper cells with CD4+ expression are categorized into Th1, Th2, Th17, and T follicular helper cells^91^,^92^. Th1 cells, which perform a vital function in combating intracellular pathogens, and Th2 cells, which safeguard against extracellular parasites and regulate inflammation through the secretion of cytokines including IL-4, IL-5, IL-6, and IL-13, have been implicated in autoimmune disorders^93^. Additionally, Th2 cells contribute to anti-inflammatory responses by secreting IL-10 and stimulate B cells to facilitate their maturation to memory and plasma cells^94^. CD8+ T cells, which exhibit cytotoxic activity and protect against intracellular pathogens, are classified as Tc1 and Tc2 subtypes based on their cytokine secretion profiles^95^,^96^. The diverse roles played by T cells in adaptive immunity emphasize the requirement of an in-depth investigation into individual T cell subtypes. Notable age-related changes have been reported in CD8+ T cells^3^. In particular, CD8+ T cells undergoing senescence exhibit a reduction in the expression of CD28 family receptors^97^. Conversely, there is a notable upregulation in the expression of immune biomarkers including CD57, TIM-3, KLRG-1, and CD45RA^98^-^100^. The augmented expression of inhibitory receptors on the surface of senescent T cells denotes a state of exhaustion, reminiscent of senescence yet governed by distinct mechanisms and characterized by unique features.^101^,^102^. In contrast to cellular exhaustion, cellular senescence is considered an irreversible process. Most of the existing research on T cell senescence focuses primarily on peripheral blood T cells, comprising a small fraction of the total T cell population^103^. Therefore, further investigation is imperative to advance comprehension of the mechanisms governing T cell senescence.

The interplay between metabolic and epigenetic factors emerges as a crucial determinant in regulating the differentiation pathways of T cells^3^,^104^. Initially, pre-T cell receptors (TCRs) are subject to genetic rearrangement in the thymus^105^. Following the antigen recognition by professional APCs, quiescent T cells undergo activation, characterized by their proliferation and differentiation into effector cells^106^,^107^. A hallmark alteration in T cells is the upregulation of aerobic glycolysis, driven by an increased expression of glucose transporters (GLUTs) on the cell surface. The escalation observed is attributed to the overexpression of genes associated with glycolysis, facilitated in a c-Myc-dependent fashion^108^. This metabolic change is accompanied by the activation of signaling cascades that contribute to the elevation of mitochondrial biogenesis and replication of mitochondrial DNA (mtDNA), which encodes factors essential for oxidative phosphorylation (OXPHOS). However, the precise underlying mechanisms remain unclear^109^. Studies have described a metabolic profile that reflects restructuring of the mitochondrial proteome, a phenomenon termed "one-carbon metabolism"^107^. In response to a viral challenge, naïve T cells rapidly multiply and diversify into specialized subsets that are important for the clearance of pathogens, destruction of infected cells, and overall management of infection^110^,^111^. Following the clearance of antigens, the predominant fate of activated T cells entails programmed cell death, known as apoptosis, with a minority transitioning into memory cells.^112^. Research indicates that CD28 interacts with various factors to activate the PI3K-PKB/AKT-mTOR signaling pathway, a critical process for TCR reorganization and the metabolic reprogramming of fatty acids, glutamine and glucose during T-cell maturation^113^,^114^. The mammalian target of rapamycin (mTOR) complexes, mTOR complex 1 (mTORC1) and mTORC2, serve as principal regulators of gene expression, each playing a unique role in the differentiation of T cells^115^. Continuous activation of mTORC1 expedites the transition of naïve T cells into CD8+ effector cells, while suppression of mTORC2 correlates with the generation of CD8+ memory T cells^116^. Senescence in T cells is characterized by a gradual transition toward a dysfunctional state, accompanied by alterations in systemic equilibrium and a general state of immunosuppression. This transition involves metabolic reprogramming and epigenetic changes, resulting in cellular aging and potential apoptosis^117^. Age-associated thymic atrophy impedes TCR rearrangements necessary for the production of double-positive thymocytes. The increased glycolytic activity of aging T cells can be partially attributed to variations in CD28 expression. During T cell aging, stress can lead to a failure to express essential components of the electron transport chain (ETC) and the subunits of OXPHOS^118^, leading to an increase in reactive oxygen species (ROS) levels; a decline in proteasomal function; and the subsequent suppression of mitochondrial biogenesis, one-carbon metabolism, and basal lipid metabolism^119^-^121^. *In vivo*, T cells in a senescent state exhibit a diminished expression of key functional molecules, including senescence-associated β-galactosidase (SA-β-gal), granzyme B (GZMB), and perforin, which are essential for cytotoxic activity^122^,^123^.

Stimulation of T cells initiates gene expression alterations that are important for driving their maturation to effector cells and maintaining their effector functions. This dynamic process is closely related to epigenetic changes. Enhancer-binding protein 4, a transiently expressed product of the c-Myc gene, serves as a crucial target of multiple molecules encoded by genes induced upon activation, playing an indispensable role in maintaining T-cell activation^124^. Effector function-associated genes are characterized by dynamic epigenetic modifications that enhance the precision and speed of their transcriptional responses to subsequent stimuli. This epigenetic imprint is preserved in human CD8+ memory T cells for an extended period and may be responsible for maintaining their effector functions^125^,^126^. T-cell differentiation is characterized by the deposition of methyl groups on the promoters of genes that are suppressed and the removal of these groups from the promoters of genes that are activated^127^. Studies employing chromatin immunoprecipitation have demonstrated that the simultaneous presence of the active mark, that is, trimethylation of histone H3 at lysine 4, and the repressive mark, that is, trimethylation of histone H3 at lysine 27, at promoter regions is a hallmark of the differentiation of naïve CD8+ T cells^128^. The degree of methylation and the extent of variation within the methylome strongly rely on the specific T cell subset, with senescent CD8+ T cells exhibiting more pronounced changes. Alterations in the global patterns of DNA methylation are considered one of the most definitive epigenetic modifications associated with senescence in T cells^129^. Usually, CpG islands within silent genes are hypermethylated and exhibit an accumulation of repressive histone modifications, whereas DNA sequences adjacent to CpG islands represent hypomethylated regions associated with senescence. Inversely correlated with the expression of genes associated with cellular immune responses and differentiation within CD8+ T cell subsets are the methylation statuses, particularly for CD27 and SATB1. This correlation suggests that epigenetic aging is linked to a reduction in T cell functionality^130^. Furthermore, in several studies, the rate of biological aging has been correlated with the degree of DNA methylation at particular CpG sites^131^. Additionally, various aging models have documented a decrease in the expression of fundamental histone proteins^132^. Senescent T cells are defined by unique patterns of chromatin condensation, termed senescence-associated heterochromatic foci (SAHF), along with increased chromatin accessibility and decreased expression of linker histone H1^133^,^134^. In combination with changes in histone modifications, hypomethylation, especially in repetitive genomic sequences situated within heterochromatic domains, could instigate genomic instability and precipitate early cellular senescence^135^,^136^. The decline in naïve T cell proportions with aging is notably correlated with a significant reduction in miR-92a expression within CD8+ T cells^130^. In senescent T cells with diminished CD28 expression, upregulation of miR-24 results in reduced expression of the histone variant H2AX, thereby impairing the cellular response to DNA damage^137^. Epigenetic modifications are crucial in driving T cell senescence and are similarly observed in the genomes of various other immune cells. Remarkably, T cells in a senescent state display epigenetic characteristics akin to those found in exhausted T cells. In a study, an in-depth analysis of tissues from different organs of mice revealed a clonal subset of CD8+ T cells expressing GZMK that exhibited a phenotype indicative of exhaustion^138^. The proportion of GZMK+CD8+ effector memory (EM) cells in human peripheral blood mononuclear cells (PBMCs) increases with age, with these cells sharing specific epigenetic changes and exhaustion markers with mouse GZMK+CD8+ T cells^138^. Basic leucine zipper ATF-like transcription factor, which is highly expressed in senescent CD8+ T cells, is crucial for differentiation leading to T-cell exhaustion^139^. Terminally exhausted T cells often display augmented chromatin accessibility within the promoters of effector genes such as GZMB and IFN-γ. This accessibility remains unaffected by aging, indicating that the mechanisms driving senescence and exhaustion share some common pathways^139^. In the natural aging process of mice, there is a progressive buildup of CD4+ T cells that exhibit a memory phenotype, characterized by the presence of the programmed cell death protein 1 (PD-1). These cells emerge as the predominant subpopulation of T cells as the mice advance in age. However, this process is markedly accelerated in mice with leukemia^140^. The expression of CD39 during aging results in a decreased proportion of CD4+ T cells^141^. In addition, accumulation of Tregs increases with age^142^-^144^, with the proportion of CD4+CD25+ Tregs in peripheral blood being higher in older mice^145^. Similarly, Treg infiltration is more pronounced and Foxp3 mRNA expression is higher in older Lewis lung cancer mouse models and older patients with lung cancer than their respective younger counterparts^146^,^147^.

## Exercise and the immune system

Physical exercise can enhance immune function by alleviating the negative outcomes of aging, including immunosenescence and inflammaging, and obesity, including inflammation and immunosuppression^9^,^148^,^149^. In addition, physical exercise can induce notable alterations in both innate and adaptive immune systems (Fig. 1). The dual role of exercise in ameliorating immune dysfunction and reshaping immune responses highlights the complexity of physiological adaptations that occur in response to physical exertion.

### Innate immune cells

#### Neutrophils

Engaging in a single session of physical exercise can substantially influence both the quantity and distribution of circulating neutrophils^150^. High-intensity resistance exercise has been shown to elevate neutrophil counts, which reach a 3-fold increase after exercise^151^-^153^. In addition, extended periods of endurance exercise, lasting from 0.5 to 3 hours, can lead to a 5-fold increase in neutrophil counts^154^. Although an increased count of neutrophils, along with that of other immune cells, is typically associated with infection and inflammation, it is noteworthy that immune cell counts induced by physical exercise usually revert to baseline levels within 6-24 hours after the cessation of exercise^155^.

Research examining the impact of physical activity on leukocytes, particularly neutrophils, has revealed that consistent exercise does not result in notable alterations in the peripheral blood leukocyte levels^156^. Studies focusing on endurance exercise have reported a notable reduction in neutrophil counts after therapeutic exercise sessions, particularly in individuals with chronic inflammatory conditions. In addition, this reduction has been associated with an increase in insulin sensitivity, body mass index (BMI), maximal oxygen consumption rate (VO2max), and fasting triglyceride levels^157^. Whether these changes are adverse or advantageous depends on the specific context. Notably, a study on resistance exercise showed that variations in the proportion of circulating neutrophils manifested more rapidly after a higher-volume/lower-intensity regimen (5 sets of 10 repetitions at 80% of one-repetition maximum, 1 RM) than after a lower-volume/higher-intensity regimen (15 repetitions at 100% of 1 RM)^158^. The reasons for the varying temporal responses of neutrophils to different exercise regimens remain unknown and warrant further investigation.

The initial increase in blood neutrophil counts after exercise is rapid and pronounced, with a subsequent, delayed increase occurring several hours after exercise. This dual-mode reaction has been associated with the extent and intensity of the physical exercise undertaken^155^,^159^. Neutrophilic leukocytosis observed promptly and subsequently after exercise is thought to be regulated by catecholamines and cortisol, respectively^160^. The ability of neutrophils to adhere to endothelial cells is an important early step in their translocation to sites of infection or trauma. However, studies have indicated that although intense, acute physical activity can enhance the chemotactic and phagocytic activities of neutrophils, it does not appear to increase their adherence to the endothelium^161^,^162^. A single session of intense exercise may attenuate the oxidative response and degranulation of neutrophils upon exposure to bacterial agents, an impact that can be sustained for a long duration. In addition, exercise can trigger an increase in the spontaneous phagocytic activity, degranulation, and oxidative burst potential of neutrophils^163^-^165^. Altogether, these findings suggest that acute physical exercise not only transiently decreases the responsiveness of neutrophils to environmental triggers but also facilitates the migration of highly active neutrophils into circulation and enhances their autonomous degranulation^166^. Although numerous studies have examined the effects of both acute and chronic exercise on neutrophils, the precise effects of exercise on the functionality of neutrophils warrant further investigation.

#### Monocytes/macrophages

Regular engagement in physical activity has been shown to reduce the baseline proportion of circulating inflammatory monocytes, particularly CD14lowCD16+ cells. Studies examining the impact of exercise through cross-sectional and longitudinal methodologies have consistently shown that participants engaging in physical exercise demonstrate a diminished proportion of inflammatory monocytes, concomitant with reduced TLR4 expression on cellular membranes and a mitigated inflammatory reaction to LPS stimulation.^167^-^169^. The precise mechanisms underlying the anti-inflammatory effects of exercise on tissue-resident monocytes remain elusive. However, preclinical studies on mouse models have shown that exercise can elicit inflammatory responses in peritoneal macrophages, suggesting that physical exercise has different effects on circulating and tissue-resident monocytes^170^-^172^. In addition, studies have shown that regular exercise can mitigate systemic inflammation in mice with high-fat diet-induced obesity^173^,^174^ and reduce macrophage accumulation at sites of chronic inflammation^175^. These findings collectively validate the anti-inflammatory effects of regular physical activity.

After exercise, the proportion of circulating inflammatory monocytes transiently increases, typically reverting to pre-exercise levels during the recovery period^176^. This acute elevation, lasting approximately 2 hours, is thought to reflect the transfer of monocytes from the marginal pool to the blood^177^. Intense exercise preferentially stimulates the release of CD14+CD16+ monocytes, which produce stronger inflammatory responses than CD14+CD16- monocytes^178^,^179^. CD14+CD16+ cells, which are capable of tissue infiltration, are released from the endothelium in response to physical exertion. The proportion of CD14+CD16+ monocytes typically decreases as the body recovers, possibly owing to their recruitment to tissues or remarginalization^176^. Furthermore, the cytokine secretion profile of monocytes is altered after exercise, with a significant decrease being observed in the expression of IL-6, IL-1α, and TNF-α, potentially owing to the downregulation of TLR on monocytes^180^-^183^. A similar immediate increase in the proportion of circulating monocytes is observed after resistance training. Based on the intensity and duration of the workout, the cell count may revert to normal within 15-30 minutes after exercise or peak 120 minutes after the cessation of exercise^151^,^152^,^158^.

Given the low frequency of circulating macrophages and their primary maturation within tissues, studies investigating the relationship between acute exercise and macrophages in humans are limited. In the tumor microenvironment, macrophages are generally characterized by an M2 phenotype, which can inhibit the effect of chemotherapy and radiotherapy, and promote tumor growth and metastasis. Tumor-associated macrophages (TAMs) of the M2-like phenotype exhibit the most pronounced ability to internalize glucose within the tumor microenvironment. This elevated glucose uptake activates the hexosamine biosynthetic pathway, thereby catalyzing O-GlcNAcylation, which is essential for the enhancement of tumor dissemination and the development of chemotherapy resistance^184^. Engaging in physical activity has the potential to preferentially polarize TAM towards an M1 phenotype, which is associated with antitumor effects. This M1 polarization can enhance the immune response against tumors, promoting the destruction of cancer cells and limiting tumor progression. A sedentary lifestyle, in contrast, may promote the skewing of tumor-associated macrophages (TAMs) towards the M2 phenotype, thereby supporting tumor progression, enhancing local invasion, and fostering metastasis to distant organs. Recent studies indicate that TAM may not be strictly M2; instead, they could represent a more complex transition from M1 to M2 as tumors progress and trigger various molecular signaling pathways^185^. Initially, M1 macrophages may be polarized in early-stage tumors. As the tumor grows and becomes malignant, these M1 macrophages could subsequently shift towards an M2 phenotype, evolving into pro-tumor "tumor-educated" macrophages.

Regular engagement in aerobic exercise is known to foster a macrophage phenotype that is anti-inflammatory within the adipose tissue of obese individuals. However, this effect is abrogated upon the specific elimination of PPAR-γ in the myeloid lineage of murine models, underscoring the pivotal function of PPAR-γ in modulating the metabolic and phenotypic adaptations of macrophages in response to aerobic exercise regimens. Deficient in PPAR-γ within myeloid cells, slender mice display intensified pro-inflammatory traits^186^. An 8-week regimen of moderate aerobic exercise on a treadmill, characterized by continuous activity at 70% of maximum oxygen uptake (Vo2 max), elicits an anti-inflammatory profile specifically in adipose tissue-resident macrophages, without affecting the phenotype of peritoneal macrophages. This observation implies that the influence of aerobic exercise training on macrophage polarization and metabolic function is inflammation-level dependent and site-specific. A plausible mechanism by which such exercise modulates macrophage polarization is via the secretion of catecholamines^187^,^188^. Monocytes and macrophages alike exhibit adrenergic receptors (ARs) and are capable of responding to catecholaminergic stimulation. During physical activity, the secretion of adrenaline elicits immunomodulatory actions, enhancing the reactivity of pro-inflammatory macrophages in healthy individuals and triggering an anti-inflammatory profile in those with elevated baseline inflammatory levels. Nevertheless, the underlying molecular pathways responsible for these immunomodulatory effects remain to be fully elucidated. Animal studies have shown that sustained exercise can reduce the antigen-presenting ability of macrophages and downregulate MHC II molecules on their surface^189^,^190^. Acute exercise can significantly enhance the phagocytic activity, nitrogen metabolism, chemotaxis, antitumor activity, and ROS levels of both M1 and M2 macrophages^191^,^192^. In addition, exercise can promote the polarization of adipose tissue-resident macrophages from the M1 to the M2 phenotype in mice, improving muscle adaptation and reducing body fat percentage. This finding indicates that increasing the proportion of M2 macrophages in adipose and muscle tissues may yield therapeutic benefits in cancer^193^.

Studies have shown that immunotherapy can regulate macrophage polarization. TAMs abundantly express both PD-1 and PD-L1, and the involvement of the PD-1/PD-L1 pathway in TAM-driven tumor immune evasion has been comprehensively demonstrated^194^. Hartley *et al.* discovered that PD-L1 consistently sends inhibitory signals to TAMs, leading to the development of an immunosuppressive M2 phenotype in these cells^194^. A separate study focusing on colon cancer showed that PD-1 has an immunosuppressive effect on TAMs by changing their phenotype and functions, potentially promoting a shift towards a tumor-promoting (M2) phenotype. Within the context of TAM alterations influenced by the tumor microenvironment, polarization has been identified as a crucial factor in the development of cancer, as it dictates the characteristics and activities of TAMs within this environment^195^.

The effect of exercise and immunotherapy on macrophage polarization is a complex process involving multiple signaling pathways and molecular mechanisms. Exercise combined with immunotherapy may enhance the therapeutic effect by modulating the polarization of TAMs. For example, exercise can promote the transformation of M2-TAM to M1 phenotype, while inhibition of SHP2 can block CD47-SIRPa pathway, restore phagocytic activity of M1 macrophages, repolarization TAM to M1 phenotype, restore its phagocytic function, promote CTLs infiltration in tumors, lead to immunostimulating TME, and significantly inhibit tumor growth^196^.

The relationship between physical activity and macrophage dynamics within the tumor microenvironment remains ambiguous, particularly regarding whether regular exercise or training reduces the infiltration of macrophages into tumors. Understanding the nuances of this phenotypic transformation and its implications for the TME is crucial and necessitates further research. Investigating how different types of physical activity influence macrophage polarization and activity could uncover new therapeutic strategies to enhance antitumor immunity and improve outcomes for cancer patients. This area of study holds promise for elucidating the complex interactions between lifestyle factors and immune responses in the context of cancer^197^.

#### Dendritic cells

A single session of dynamic exercise can lead to an increase in the production of monocyte-derived DCs in healthy adults; however, the functional consequences of this effect remain unclear^198^. The post-exercise increase in the number of circulating DCs may not always result in a heightened inflammatory response^199^,^200^. Preclinical studies have shown that consistent exercise can lead to a pronounced increase in the mixed leukocyte reaction, the surface expression of MHC II molecules, and the production of IL-12 by DCs. However, no significant differences are observed in the expression of costimulatory molecules, such as CD80 and CD86, on DCs before and after exercise^201^,^202^. Aerobic exercise may preferentially activate pDCs, which produce IFNs to counteract viral and bacterial infections, and enhance immune surveillance through the selective stimulation of pDCs^203^. Despite these findings, the effects of exercise on DCs remain elusive, necessitating further investigation.

#### NK Cells

NK cells have emerged as a major research hotspot in the field of exercise immunology owing to their notable modifiability by physical activity and the relative simplicity of their examination^204^,^205^. Multiple studies have validated the effects of exercise on both functionality and abundance of NK cells; however, the findings remain controversial. Similar to other leukocytes, NK cells are swiftly released into the bloodstream following acute physical exercise. This release is enhanced by catecholamines' effects on adhesion molecules and the consequent rise in shear stress^204^-^206^. NK cells preferentially adhere to human endothelial cells (EC). Catecholamines, acting through β2-ARs, are capable of facilitating the mobilization of NK cells from the marginating reservoir to the circulating compartment. This process is mediated by altering the adhesive interactions between NK cells and EC. However, protracted periods of exercise may lead to a decrease in the proportion of circulating NK cells owing to their migration to tissues or their remarginalization^204^,^205^. High-intensity resistance training, involving 60-100% of one-repetition maximum (1 RM) across various volumes, can result in a transient increase in NK cell counts, with this increase lasting up to 15 minutes after exercise^151^,^152^. Additionally, the proportion of CD16+/CD56+ NK cells reverts to the baseline level within 3 hours after a session of sustained aerobic exercise^153^.

Variations in the proportion of CD16+/CD56+ NK cells have been positively associated with the intensity and extent of the physical activity undertaken. Notably, in contrast to other lymphocyte subsets, CD16+/CD56+ NK cells remain unaltered after low-intensity resistance training^207^. NK cells are characterized by their innate cytotoxic activity, which involves the secretion of IFN-γ and promotion of apoptosis in infected or transformed cells. The cytotoxic potential of NK cells is an important measure of their functional status^208^. Early-stage and cross-sectional studies have indicated that a session of moderate-intensity exercise can enhance the cytotoxic activity of NK cells^209^,^210^, which typically decreases as the body enters the recovery phase^211^. These fluctuations in cytotoxic activity can be primarily attributed to changes in the relative frequency of NK cells in the PBMC population. Both high- and moderate-intensity exercises have been associated with notable changes in the proportion of circulating NK cells, which can markedly affect the interpretation of NK cell cytotoxicity assessment^211^. However, studies employing multiple target tumor cells (e.g., K562) to examine the impact of exercise on the cytotoxic activity of NK cells have proposed an alternative perspective^212^. In particular, these studies have suggested that exercise leads to selective re-distribution of NK cell subsets exhibiting a mature phenotype, which augments their cytotoxic activity against targets expressing HLA^212^,^213^. Consequently, the nature of the changes observed in NK cell functionality in the context of exercise remains ambiguous. Whether these changes are exclusively indicative of variations in the quantity and distribution of NK cell subpopulations induced by exercise or whether exercise affects the intrinsic functionality of individual NK cells remains unclear.

### Adaptive Immune Cells

#### B Cells

The effects of physical exercise on humoral immunity, specifically the functionality of Igs, have been evaluated by measuring Ig levels in both mucosal and systemic environments. Studies investigating the impact of exercise, whether short- or long-term, on Igs have shown that systemic Ig concentrations are either moderately increased or remain unchanged in response to physical exertion^214^,^215^. The mucosal immune system protects the mucous membranes in the respiratory, nasal, and gastrointestinal tracts. This protective effect is mediated by the secretion of secretory immunoglobulin A (SIgA) by specialized plasma cells. SIgA is strategically positioned to identify and neutralize pathogens that threaten the mucosal barriers, thereby serving as the first line of defense against pathogen invasion^216^,^217^. Extensive efforts have been directed towards understanding how physical activity influences the concentration of sIgA in saliva^218^. Studies have demonstrated that the structured program of an exercise regimen and the intensity and duration of the exercise are key factors affecting the concentration of sIgA^8^,^219^. Elevation in salivary sIgA levels signifies bolstered immune functionality and correlates with reduced susceptibility to upper respiratory tract infections (URTIs) among those embracing an active physical regimen. On the contrary, a significant temporary decrease in salivary SIgA levels can increase susceptibility to URTIs^220^,^221^. Although earlier studies have shown that athletes participating in endurance activities have a decreased salivary concentration of sIgA, especially during rigorous training^221^-^223^, several recent studies have indicated that the salivary concentration of SIgA in athletes is usually similar to that in non-athletes, except under conditions of high-intensity exercise^224^,^225^. The post-exercise decrease in salivary sIgA levels is attributed to the diminished inhibitory effect of the parasympathetic nervous system to some extent^219^. Consequently, moderate-intensity exercise sessions have a negligible impact on the expression of Igs in plasma cells, whereas prolonged and vigorous exercise can decrease the salivary concentration of SIgA. The number of B cells, which are crucial for Ig synthesis, may slightly increase during and immediately after exercise based on the duration and intensity of the exercise. However, the increased B cell count typically decreases below the pre-exercise level during the initial stages of recovery and returns to the baseline level within a day^155^,^226^. Consistently, studies have reported a higher proportion of circulating B cells during or after high-intensity resistance training, with the effects persisting after a 3-hour recovery period and across various volumes of training sessions performed at 60-100% of 1 RM^153^,^227^,^228^. Additionally, low-intensity resistance training has been shown to increase the proportion of circulating B cells^229^. It is noteworthy that physical exercise of different intensities can induce acute lymphocytosis, either during or immediately after the activity. In-depth research is warranted to understand the impact of physical exercise of various intensities on B cell counts in the bloodstream and to elucidate the influence of exercise regimens on the functional aspects of B cells.

#### T Cells

Numerous studies have shown that T cell proliferation is suppressed after physical exertion^4^,^8^. In athletes who undergo high-intensity training, the functionality of T cells, particularly circulating Th1 cells, is highly sensitive to increased training volumes. Protracted periods of intense training decrease the proportion of circulating Th1 cells, compromising their functionality. However, lymphocytosis is common, marked by an increase in T cell counts during and immediately after exercise, followed by a decline below the baseline level in the early recovery phase^230^,^231^. Modulation of T cell counts in response to various exercise regimens usually relies on the intensity and extent of the physical activity undertaken^230^,^231^.

Regarding resistance training, the post-exercise response of CD4+ T cells varies among different study cohorts. The number of CD4+ T cells increases immediately after high-intensity resistance training^151^,^152^,^227^ and within 1 hour after low-intensity resistance training^232^. It typically reverts to the pre-exercise level within 30 minutes after high-intensity exercise^152^ but remains high for up to 1 hour after low-intensity exercise^232^. Despite an overall increase in total lymphocyte counts, no significant changes in CD4+ T cell counts are observed before and after moderate-intensity resistance training (60-70% of 1 RM across various volumes)^153^. In the resting phase (>24 hours after the last training session), the proportion of circulating lymphocytes, encompassing all T cell types, in athletes is usually comparable to that in non-athletes^233^.

Similar to the number of CD4+ T cells, that of CD8+ T cells transiently increases after high-intensity resistance training (aligning with intensities of 60-70% of 1 RM), reverting to the baseline level within the first 15 minutes of the recovery period or decreasing below the baseline level within the first 30 minutes of the resting phase. Complete restoration of CD8+ T cell counts is usually achieved within 3 hours after the cessation of exercise^152^,^153^,^227^. Low-intensity resistance training has been shown to induce an increase in the proportion of CD8+ T cells, which begins during the initial phase of the activity and persists for up to 60 minutes after exercise. The increased proportion of CD8+ T cells tends to normalize and return to the pre-exercise level within 20-60 minutes after the cessation of exercise^232^. Differences in exercise regimens and the timing of post-exercise blood sampling may account for the inconsistency in CD8+ T cell counts across studies. While the precise impact of physical exercise on T cell proliferation, both during and post-exercise, remains elusive, it is evident that exercise instigates alterations in T cell behavior. Therefore, in-depth studies are warranted to understand the correlation between the number and function of T cells in the context of different exercise regimens.

## Exercise modulation of immunosenescence and anti-tumor immunity

### Immunosenescence and cancer

**Figure 2:**
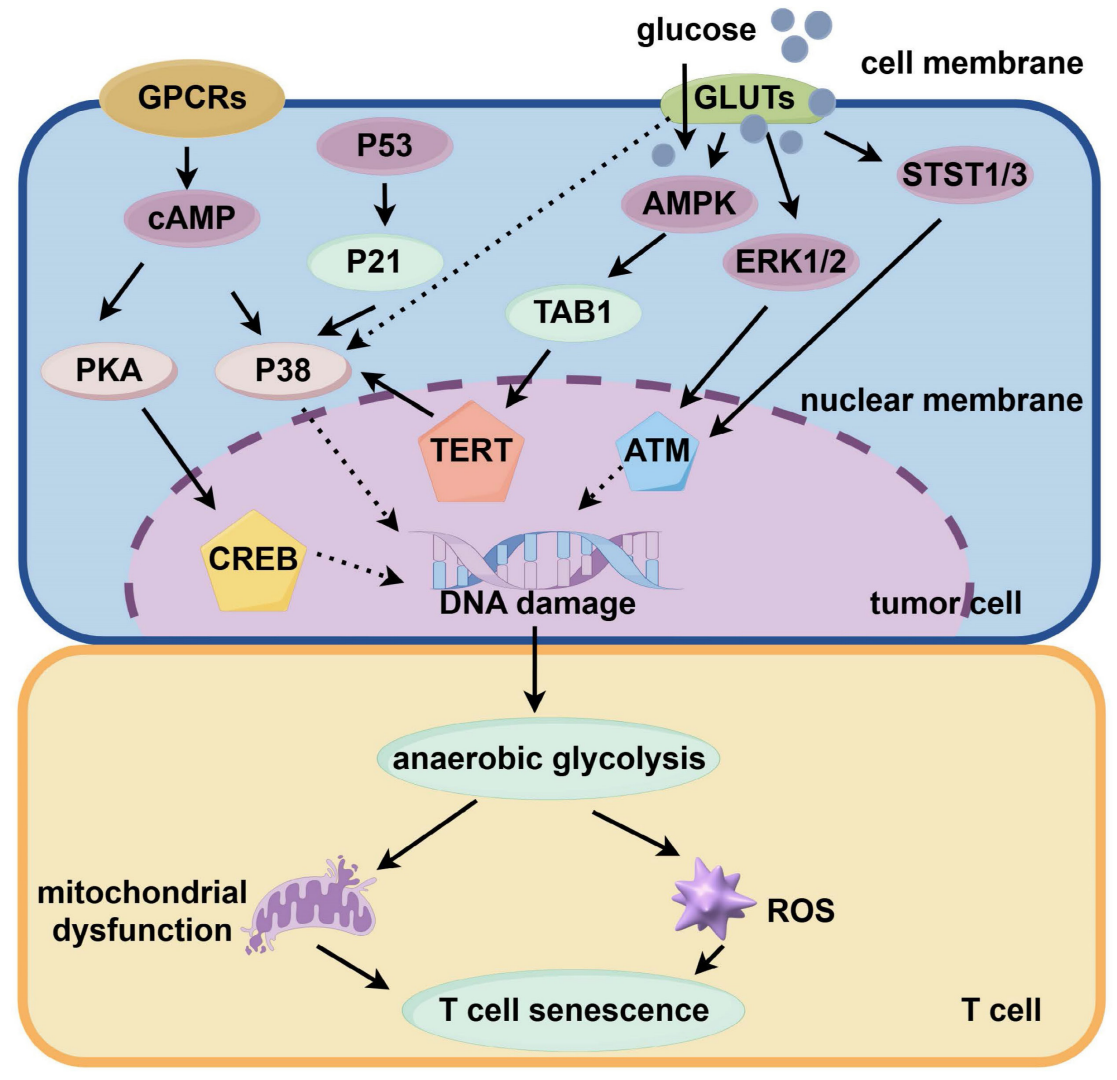
** Several factors can trigger immune cell senescence, impairing cellular function.** Tumor cells increase cAMP levels, which induce T cell senescence through PKA-CREB and P38 signaling pathways, leading to DNA damage. Glucose competition triggers DNA damage via the ATM pathway, activating ERK1/2, P38, and STAT1/3 pathways, ultimately halting T cell cycles. AMPK activation exacerbates P38 activity, suppresses telomerase reverse transcriptase gene expression, and promotes anaerobic glycolysis in senescent T cells, causing mitochondrial dysfunction and increased ROS production. GPCR: G protein-coupled receptor; cAMP: cyclic adenosine monophosphate; PKA: cyclic-AMP dependent protein kinase A; CREB: cAMP-response element binding protein; TERT: telomerase reverse transcriptase; GLUT: glucose transporter; AMPK: AMP-activated protein kinase; TAB1: TGF-Beta activated kinase 1; ERK: extracellular signal-regulated kinase; ATM: ataxia-telangiectasia mutated.

The incidence of malignant neoplasms increases with age. This positive correlation is governed by two mechanisms as follows: progressive accumulation of genetic aberrations and immunosenescence, which is a key factor in carcinogenesis (Fig. 2). The geriatric population has been shown to have an increased risk of developing neoplasms^234^. In a study, a meticulous assessment of mammary epithelial cells from women aged between 16 and 91 years demonstrated that the age-related progressive accumulation of luminal and stem cells significantly increased the risk of malignant tumors^235^. This finding highlights the close relationship between aging, cellular behavior, and the risk of cancer.

In the complex and dynamic TME, various factors can induce a state of senescence in immune cells, leading to the impairment of cellular function. The role of immunosenescence in tumors is complex, involving various factors such as cAMP, glucose competition, and oncogenic stress within the tumor microenvironment. These factors can lead to the senescence of T cells, macrophages, natural killer cells, and dendritic cells^1^. In the geriatric population, immune dysfunction not only contributes to the initiation of tumors but also accelerates the aging of T cells. Immune system disorder in TME is characterized by an increased proportion of tumor-associated macrophages and Tregs^236^-^238^. Under hypoxic conditions, tumor cells exhibit an endogenous elevation in cyclic adenosine monophosphate (cAMP) levels, which serves as an intrinsic factor facilitating T cell senescence by attenuating the functionality of tumor-specific effector T cells^239^,^240^. Furthermore, tumor cells can initiate the PKA-CREB and P38 signaling pathways by generating cAMP, consequently leading to DNA damage and triggering T cell senescence^40^,^236^,^241^,^242^. Competition for glucose can induce DNA damage via the ATM pathway, triggering activation of the ERK1/2 and P38 pathways. This collaborative effect with STAT1/3 ultimately halts the T cell cycle, leading to senescence. Glucose-induced activation of the ATM and AMPK pathways promotes DNA damage, driving T cell senescence. AMPK further amplifies P38 activation by interacting with TAB1 protein, while concurrently suppressing the expression of the telomerase reverse transcriptase gene^18^,^243^. P38 has been shown to impede cell cycle progression by activating P53, P21, and P16^18^. T cells undergoing senescence increasingly rely on anaerobic glycolysis as their primary source of energy. This metabolic shift can lead to mitochondrial dysfunction and consequently increase ROS production^242^,^244^. Furthermore, the NFκB, C/EBPβ, and cGAS-STING signaling pathways have been shown to play an important role in T cell senescence^3^,^90^. Tumor cell stimulation via TLR8 activation has been demonstrated to bolster antitumor immunity through dual mechanisms: hindering senescence advancement in both senescent T cells and tumor-specific ones, thereby alleviating their immunosuppressive influence, both *in vitro* and *in vivo*^242^.

Multiple preclinical and clinical studies (NCT04924374, NCT05807243, NCT04772092) have investigated the consequences of immunosenescence. Preliminary findings suggest that immunosenescence, particularly its impact on CD8 T cells, plays a crucial role in the development and management of breast cancer^245^. Studies using murine models of breast cancer have reported a decline in IFN signaling in CD8 T cells of older animals^246^. The senescent cellular environment is closely related to tumor metastasis and invasion, with T cell metabolism in TME exhibiting age-related changes. Compared with younger patients with melanoma, older patients exhibit more pronounced ECM alterations owing to changes in hyaluronan and proteoglycan link protein 1 (HAPLN1), which can enhance the invasiveness and metastatic potential of melanoma cells^247^. Additionally, the age-dependent increase in the secretion of sFRP2 by fibroblasts has been associated with enhanced angiogenesis and metastasis in melanoma^248^. Notably, senescence can have beneficial effects on the outcome of cancer. In several mouse models of tumors, including MC38, B16, and 4T1, a slower rate of tumor growth has been observed in older mice^249^. Elderly individuals with bronchial cancer have been shown to have reduced tumor velocity and metastasis, possibly owing to host factors associated with aging that may limit the proliferation and spread of aggressive tumor cells^250^. Similarly, older individuals with Engelbreth-Holm-Swarm cancer and B16F10 melanoma mouse models have been shown to have decreased tumor growth and metastasis along with increased survival rates^251^,^252^. A historical review of 1869 breast cancer cases reported a higher incidence of invasive ductal carcinoma in patients aged <39 years^253^. Moreover, recent research has demonstrated an increase in serum methylmalonic acid levels among elderly individuals, resulting in the upregulation of SOX4 expression. This molecular event may trigger transcriptional reprogramming, consequently enhancing the aggressiveness of cancer cells^254^. These diverse findings collectively emphasize the complex relationship between aging and cancer development.

The influence of chemotherapy on senescence of CD8 T cells in breast cancer has been demonstrated^245^. In a preclinical pancreatic ductal adenocarcinoma model, T/P drug treatment has been shown to induce senescence in pancreatic tumor cells and initiate the formation of a SASP, which is important for vascular restructuring. The process not only enhances the efficacy of chemotherapy by improving its uptake and potency but also promotes the infiltration of T cells into malignant tissues^255^. These findings suggest a correlation between the initiation and advancement of cancer and immunosenescence.

### Effects of exercise on immunosenescence

**Figure 3:**
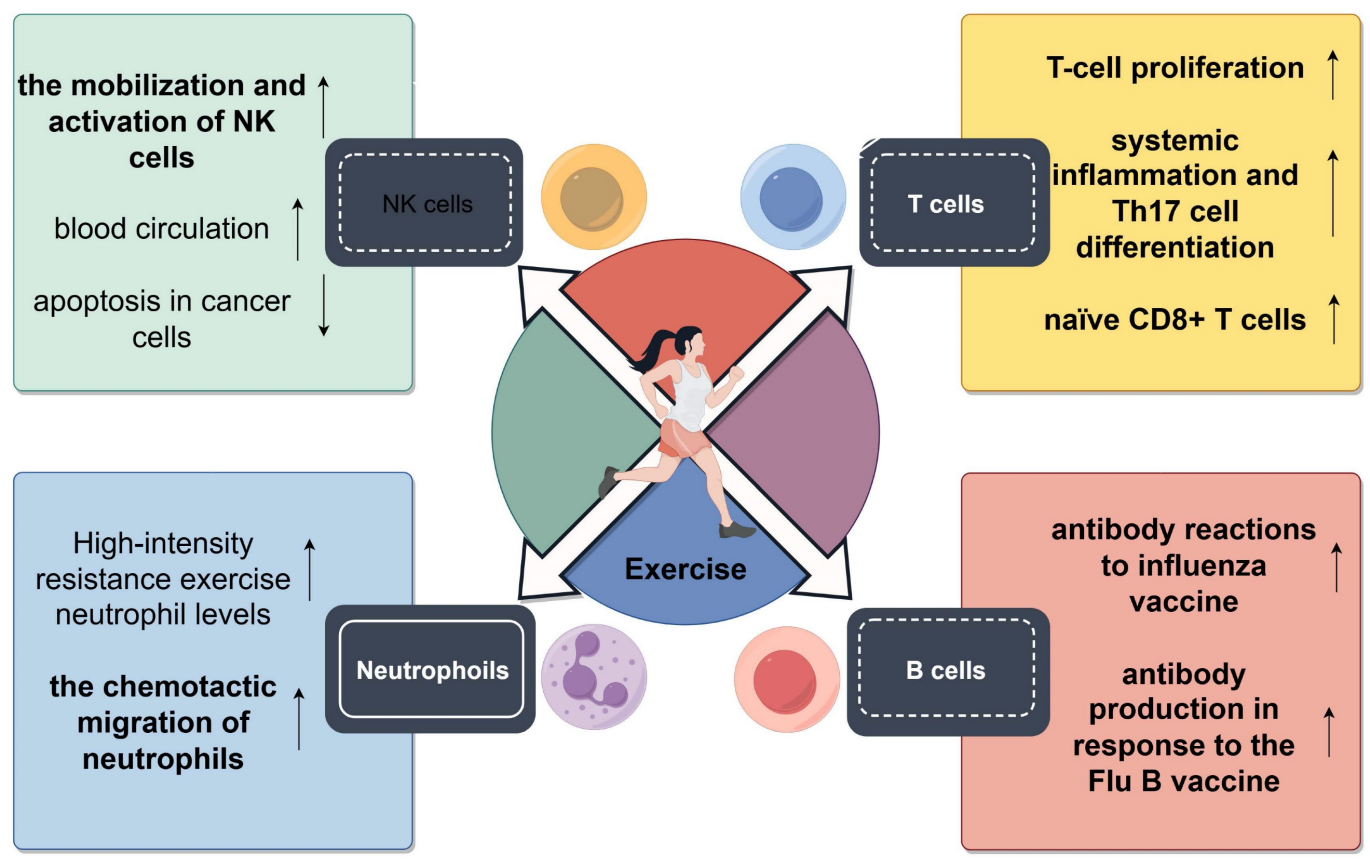
** Beneficial impact of regular physical activity on immunosenescence.** Numerous studies have highlighted the beneficial impact of regular physical activity on immunosenescence, examining both innate and adaptive immune functions. Regular physical exercise enhances NK cell activity and boosts neutrophil chemotactic response; Routine exercise in older adults correlates with enhanced T cell proliferation and reduced indicators of immunosenescence, including lower systemic inflammation and Th17 cell differentiation; Men who maintained regular physical activity showed stronger antibody responses to influenza vaccines. Older women engaging in regular physical activity exhibit enhanced antibody response to the Flu B vaccine.

Several studies have emphasized the positive influence of an active lifestyle on immunosenescence, focusing on both innate and adaptive immune components^9^,^10^ (Fig. 3). Cross-sectional studies have consistently demonstrated that older adults with increased levels of physical fitness experience more health benefits than those with a less active lifestyle. A strong correlation is observed between regular physical exercise and the increased activity of NK cells in elderly individuals^256^. Additionally, the chemotactic activity of neutrophils in response to IL-8 is high in physically active, healthy elderly individuals^257^. Interventional studies have further delineated the impact of exercise on important aspects of innate immunity. Notably, the proportion of proinflammatory and senescent nonclassical CD14+/CD16+ monocytes increases after a 12-week program involving moderate-intensity strength and endurance training^167^. In the context of rheumatoid arthritis, high-intensity interval training has been shown to increase the oxidative burst and phagocytic activity of neutrophils^258^. These findings collectively indicate that regular physical activity can enhance innate immune responses, potentially decreasing the risk of infections and alleviating inflammation.

Although the response of an aging innate immune system to physical exercise remains to be elucidated, numerous studies have examined the effects of physical exercise on the adaptive immune system, with a special focus on T cells. An early cross-sectional study showed that mitogen-induced T cell proliferation was more pronounced in highly trained older women than in less trained older women^256^. Another study reported that elderly runners with an average of 17 years of training had improved T cell proliferation, which was associated with enhanced adaptive immunity^259^. Additionally, a study on healthy, non-competitive adults aged between 55 and 79 years demonstrated that these individuals exhibited minimal indicators of immunosenescence, with their thymic function markers being comparable to those in younger individuals. These physically active individuals exhibited lower levels of systemic inflammation and Th17 cell differentiation, with no significant differences being observed in the proportion of naïve T cells and regulatory B cells between them and their peers with a sedentary lifestyle. However, the proportion of senescent CD28-CD57+ T cells was found to be similar between active and inactive older adults^260^. The study reveals a significant correlation between cardiovascular endurance and the occurrence of senescent T cells within the circulatory system. This finding suggests that elevated levels of peak oxygen uptake are linked to a reduction in the percentage of senescent T cells and an augmentation in the proportion of naïve CD8+ T cells^258^. This relationship remains significant after accounting for variables such as age, BMI, and body fat percentage. However, when adjusted for VO2max values, the correlation between age and the proportion of senescent T cells is non-significant, indicating that cardiovascular fitness may significantly influence T cell dynamics related to aging^261^. Minuzzi *et al.* examined 19 master athletes aged >40 years with extensive training backgrounds. They found a decreased proportion of senescent central memory (CM) CD8+ T cells, senescent EM CD8+ T cells, and senescent CM CD4+ T cells in these athletes when compared with inactive individuals^28^. Additionally, the athletes had a decreased proportion of highly differentiated EM-like T cells across both CD4+ and CD8+ subsets^262^. Based on these findings, Minuzzi *et al.* proposed that regular exercise may not only prevent the accumulation of the aforementioned cell types but also promote their clearance through processes such as apoptosis^263^. A subsequent study showed that intense, acute exercise sessions primarily induced apoptosis in T cells exhibiting a senescent phenotype, thus validating the aforementioned hypothesis^264^. Furthermore, there is consistent evidence from cross-sectional studies indicating that routine physical activity has the potential to avert age-associated alterations in lymphocyte subgroups and partially ameliorate the decline in T cell functionality that occurs with aging.

Findings from cross-sectional studies have been tested and validated to varying extents through controlled exercise-based interventions. For instance, a study involving overweight postmenopausal women aged 50-75 years did not observe any changes in T cell proliferation after a 12-month aerobic exercise regimen^265^. Similarly, another study showed that a 32-week exercise regimen integrating endurance and strength training did not lead to an increase in T cell proliferation in older adults^266^. On the contrary, consistent endurance training has been shown to have positive effects on the CD4+-to-CD8+ T cell ratio in elderly individuals^150^. In individuals with prediabetes, a 3-week endurance training program has been shown to increase the proportion of naïve and CM T cells while decreasing the proportion of senescent EMRA CD8+ T cells^267^. Various factors can affect these results, including the type of exercise undertaken, which may have an impact on immunosenescence. In particular, endurance training has more positive effects than strength training^268^,^269^. The initial health status of participants may also serve as a contributing factor. Although many studies have focused on sedentary but overall healthy older individuals, the findings have indicated that individuals characterized by a "healthy risk" profile or identified as having an increased risk of immunosenescence (elevated IRP) may gain more advantages from regular physical activity. For example, sustained aerobic training has been shown to improve T cell proliferation in postmenopausal women after successful breast cancer treatment^270^. Additionally, individuals with prediabetes who have a diminished health status or specific conditions that elevate their IRP may be more likely to experience a reduction in the signs of immunosenescence through engagement in regular exercise. However, further research is warranted to clarify the specific types of exercise that are effective, understand the relationship between exercise frequency and response, and examine the efficacy of physical activity in restoring immune function.

The influence of physical activity on key health metrics has been extensively investigated, with multiple studies highlighting its effects on the response to vaccination. A study showed that men aged 65-85 years who had maintained a routine of physical activity for approximately 25 years exhibited more robust antibody reactions to influenza vaccines than their less active age-matched counterparts. Mechanistically, sustaining thymic function and preserving a pool of naïve T cells played an important role in delaying immunosenescence^271^. Another study showed that an exercise regimen performed thrice weekly at a moderate intensity over 10 months led to a significant increase in antibody titers after influenza vaccination^272^. In addition, older women who engage in regular physical activity have been shown to have higher antibody production in response to the Flu B vaccine when compared with their less active age-matched peers, with the response remaining elevated 18 months after the vaccination^273^. The relationship between exercise and immunosenescence has been examined in the context of various diseases, including cancer. Given that many patients with cancer present with an elevated risk of immunosenescence (IRP) and an active lifestyle is known to decrease the risk of cancer and yield better therapeutic outcomes, it can be hypothesized that some advantages of regular physical exercise are attributable to its effects on delaying immunosenescene^274^.

### Effects of exercise on anti-tumor immunity

Immunosenescence remarkably increases the risk of cancer in elderly individuals, characterized by alterations such as decreased NK cell function, increased inflammation, compromised antigen processing by monocytes and DCs, accumulation of dysfunctional senescent cells, and reduced proportion of naive T cells capable of preventing the emergence of cancer cells. However, physical activity has been shown to delay the onset of immunosenescence. It enhances NK cell activity, strengthens antigen presentation mechanisms, attenuates inflammatory responses, and limits the accumulation of senescent cells, potentially serving as a preventive measure against immunosenescence-induced malignancies^12^,^275^.

With regard to the effects of physical activity on immune responses, NK cells exhibit the highest sensitivity to exercise-induced changes, followed by T cells, whereas B cells exhibit the least responsiveness^231^. NK cells, which are notably affected in the earliest stage of exercise, play an essential role in the innate immune system, whereas T cells are important for orchestrating the adaptive immune response. The rapid response of NK cells to exercise is attributed to their inherent reactivity and activation of specific receptors, including NKG2D and H60a, and the NKR-P1B receptor complex ligand Clr-b^276^-^278^. Pedersen *et al.* showed that adrenaline-mediated NK cell mobilization significantly increased immune cell infiltration, which prevents cancer growth, in mouse models^279^. On the contrary, inhibition of NK cell mobilization by the β-adrenergic receptor antagonist propranolol has been shown to promote cancer growth and reduce immune cell infiltration in tumors, indicating that adrenergic signaling may serve as a target for immune cell mobilization-dependent cancer suppression. Additionally, the targeted regulation of cancer cell metabolism may enhance anti-cancer immunity^280^,^281^. Integration of physical exercise with anti-cancer treatment strategies has been shown to enhance the mobilization and activation of NK cells, improve blood circulation, and promote cancer cell apoptosis, which can increase the core temperature of the body^205^. In addition, physical exercise exerts anti-inflammatory effects by reducing adipose tissue and increasing the number of anti-inflammatory Tregs, thereby alleviating systemic inflammation, promoting the release of anti-inflammatory cytokines^282^, and suppressing pro-inflammatory cytokines during muscle activity^283^. Both acute and chronic physical activity can induce cytokine responses that activate immune cells within the TME, leading to varied effects on cancer growth. The immunophenotypic landscape of the TME is dynamic and is significantly influenced by fluctuations in cytokine levels^197^.

In a study, acute physical activity was found to substantially increase the number of apoptotic lymphocytes in peripheral blood during a systemic immune response. Simultaneously, the proportion of senescent and memory T cells, including both CD4+ and CD8+ subpopulations, was remarkably increased^284^. Long-term engagement in physical activity has been shown to increase CD28 and T lymphocyte levels in both younger and older individuals^284^. Recent studies have indicated that moderate-intensity physical exercise does not influence the concentration of anti-inflammatory cytokines but may increase the levels of pro-inflammatory cytokines and enhance antigen-specific immune responses. These enhanced immune responses are associated with a decreased likelihood of developing cancer. On the contrary, high-intensity physical exercise can inhibit T cell proliferation and attenuate cytotoxic responses to specific antigens. Moreover, there exists a correlation with elevated levels of inflammatory cytokines and an augmented proportion of CD4+CD25+ Tregs, potentially heightening susceptibility to infections^285^. Although high-intensity exercise may not represent the optimal approach to preventing cancer development, moderate-intensity exercise has shown efficacy in impeding the progression of neoplastic diseases.

Altogether, physical activity can enhance immune cell mobilization and infiltration in the TME through the recruitment of NK cells, induction of cytokine-mediated anti-inflammatory responses, and amplification of T cell proliferation. These protective effects play an instrumental role in suppressing the proliferation of cancer cells and delaying the onset of immunosenescence.

### Immunosenescence and immunotherapy

#### ICIs

ICIs are a class of monoclonal antibodies that can neutralize the suppressive impact of immunoregulatory proteins on the immune responses of effector cells. In clinical settings, ICIs are used to target PD-1 and CTLA-4 owing to the established function of these checkpoints in suppressing T cell activation and proliferation. Although age is not considered a primary prognostic factor in patients receiving ICIs, selection bias is evident owing to the under-representation of older individuals in clinical trials despite the high prevalence of cancer among them. On the contrary, older patients have been shown to have lower progression-free survival (PFS) and overall survival (OS) rates than younger patients in the CheckMate-017 trial for NSCLC, CheckMate-025 trial for RCC, Keynote-045 and 052 trials for urothelial carcinoma (as monotherapy), CheckMate-067 trial for melanoma, CheckMate-214 trial for RCC, and CheckMate-227 trial for NSCLC (which involved anti-PD-1/PD-L1-based combination therapies).

Recent studies have suggested a relationship between immunosenescence and decreased effectiveness of immune checkpoint blockade (ICB) (Table. 1). The pre-treatment proportion of senescent T cells may help predict treatment outcomes. Ferrara *et al.* identified a senescent immune phenotype (SIP) in CD8 T cells characterized by the loss of CD28 and the expression of CD57 and KLRG1 in patients with advanced NSCLC^286^. An elevated systemic immune-inflammation index (SIP) before treatment, specifically >39.5%, has been associated with low PFS and OS rates in patients undergoing ICB therapy. This association is evidenced by the decreased rate of objective response to ICB, a trend that is not observed in patients undergoing chemotherapy. T cells in these patients have a decreased proliferation rate and increased secretion of inflammatory cytokines, without a discernible relationship with the age or chemotherapy history of the patients. In patients with melanoma receiving ipilimumab, a higher baseline proportion of differentiated EM CD8 T cells has been associated with a lower OS rate^287^. Similarly, in patients treated with pembrolizumab, either alone or in combination with a lower dose of ipilimumab, a higher pre-treatment proportion of CD27-CD28-Tim-3+CD57+ T cells has been associated with a lower OS rate^288^. Although non-senescent T cells are considered indispensable for effective ICB, further research is warranted to examine the effects of T cell senescence on the efficacy of ICB.

SASP is a hallmark of cellular aging characterized by an increase in the serum levels of pro-inflammatory cytokines, including IL-1, IL-6, IL-8, and TNFα. Given that these cytokines play an important role in promoting tumorigenesis, SASP may be associated with decreased efficacy of ICB. For example, IL-6 can interfere with the activation of T cells by monocyte-derived DCs in the TME^289^ and is associated with higher C-reactive protein levels and poorer outcomes in patients undergoing ICB therapy^290^. IL-1 can induce the production of CXCL1, CXCL2, and CXCL5 in intra-tumoral macrophages and monocytes, leading to the recruitment of neutrophils and MDSCs that can suppress antitumor immunity^291^. Additionally, IL-8 contributes to unfavorable responses to ICB, particularly by facilitating the recruitment of neutrophils and myeloid cells and by promoting immune evasion mechanisms involving NETs^292^.

The relationship between inflammaging and the efficacy of ICB therapy remains unclear^281^. Studies have suggested that pro-inflammatory SASP has positive effects on treatment outcomes, especially in cases in which chemotherapy or radiation therapy induces a state similar to senescence^293^,^294^. In melanoma characterized by dense immune cell infiltration, tumor cells can upregulate SASP-related genes, potentially leading to resistance to anti-PD-1 treatment^295^. However, some studies have revealed contrasting results. In lung adenocarcinoma, a senescence-associated gene expression pattern has been associated with a reduction in OS and is thought to be associated with SASP owing to the increased levels of IL-1α, IL-1β, IL-6, and VEGF^296^. Secretion of IL-6 by senescent osteoblasts can promote the formation of a microenvironment conducive to bone tumor growth^297^. Additionally, IL-6 secreted by tumor-resident senescent stromal cells can enhance MDSC infiltration and decrease the efficacy of anti-tumor T cell responses^298^. The complexity and discrepancies among the findings of existing studies pose a significant challenge to understanding the relationship between inflammaging and the outcomes of ICB therapy.

#### Adoptive T cell therapy

Chimeric antigen receptor (CAR)-T cell therapy is a well-known immunotherapeutic strategy for cancer. It involves the *ex vivo* genetic modification of patient-derived T cells to produce receptors that are capable of identifying specific antigens. These cells are expanded *ex vivo* and are reintroduced into the patient to selectively target and destroy cancer cells^299^. A major advantage of CAR over traditional TCRs is its ability to detect tumor-associated antigens without the requirement of HLA compatibility, which expands the range of target antigens.

Clinical studies have validated the therapeutic efficacy and safety of CAR-T cell therapy, particularly in hematological cancers. The FDA has approved the use of CAR-T cell therapy for the treatment of acute lymphoblastic leukemia, diffuse large B-cell lymphoma, mantle cell lymphoma, follicular lymphoma, and multiple myeloma that are refractory or have relapsed^300^-^302^. Additionally, the benefits of CAR-T cell therapy have been observed in elderly individuals with hematological cancers. At present, multiple clinical trials are investigating the efficacy of CAR-T cell therapy in solid tumors, including but not limited to ovarian cancer, HER2-positive breast cancer, EGFR-positive biliary tract cancer, gastric cancer, pancreatic cancer, hepatocellular carcinoma, and colorectal cancer^303^.

Senescent T cells are present throughout CAR-T cell therapy. In the cell collection stage, patients with diffuse large B-cell lymphoma (DLBCL) who are non-responsive to anti-CD19 CAR-T cell therapy exhibit a higher proportion of CD28-CD27-CD3 T cells in leukapheresis samples^298^. Additionally, the proportion of senescent-like CD8+CD28-CD57+CD39+ T cells is found to be increased in CAR-T cells obtained from patients with refractory or relapsed DLBCL and AML^304^. After infusion, BCMA-CAR-T cells potentially acquire a senescent phenotype characterized by the expression of KLRG1 and CD57 in patients with multiple myeloma; however, the correlation with treatment efficacy remains unclear^305^. Therefore, evaluating T cell senescence is crucial for understanding the resistance mechanisms associated with CAR-T cell therapy.

The immunosuppressive microenvironment of solid tumors may impede the efficacy of CAR-T cell therapy^306^. The use of CAR-T cell therapy for treating solid tumors is challenging owing to the presence of an unfavorable TME, the risk of on-tumor/off-tumor toxicity, and low antigen specificity^307^. A promising strategy to overcome these challenges is the combined administration of CAR-T cells and histone deacetylase inhibitors (HDACis). Ali *et al.* showed that treatment of mice with pancreatic cancer with panobinostat, an HDACi, enhanced the antitumor efficacy of CAR-T cells by driving their maturation to CM cells, thereby enhancing the epigenetic accessibility of genes related to T cell memory^308^. The use of HDACis to catalyze the phenotypic transition of endogenous T cells toward a more durable memory phenotype may have beneficial effects in cases with pronounced T cell senescence.

#### NK cell therapies

Owing to their diverse immune functions, NK cells are gaining recognition as a promising option for cellular immunotherapy^309^. The therapeutic application of NK cells has shown encouraging results in the management of hematological cancers and after organ transplantation in clinical settings^310^. Evaluating the safety and potential side effects of chemotherapy in older individuals with AML, which has a higher prevalence among elderly individuals, is a topic of considerable interest. The susceptibility of elderly patients to the toxic effects of chemotherapy substantially challenges the treatment. Therefore, using NK cell-based immunotherapy in this age group represents a promising therapeutic approach. A recent clinical study (NCT00540956) examined the post-chemotherapy recovery of NK cell function in elderly patients with AML who had achieved first remission, providing insights into the influence of chemotherapy on NK cells. Another clinical study (NCT00799799) validated the feasibility and safety of using KIR-ligand-mismatched NK cells in elderly patients with AML who had undergone immunosuppressive chemotherapy, highlighting the therapeutic potential of NK cells in this population^75^. The use of KIR-ligand-mismatched haploidentical NK cells as a consolidation therapy after the first complete remission has been associated with prolonged disease-free survival in elderly patients with AML, especially when a large number of NK cells is infused^311^.

### Exercise and immunotherapy

#### Immune checkpoint inhibitors

Recent studies on animal models have provided insights into the potential synergistic benefits of physical exercise and anti-PD-1 therapy. Wennerberg *et al.* demonstrated that integration of 30 minutes of treadmill activity daily with PD-1 blockade and radiation therapies significantly reduced the proportion of tumor-infiltrating MDSCs in breast cancer. This reduction was accompanied by increased CD8+ T cell infiltration, which effectively transformed the immunosuppressive TME into an environment more conducive to effector cell activity. Consequently, the group engaged in physical activity exhibited a pronounced decrease in tumor growth when compared with the sedentary group that received equivalent anti-PD-1/radiation therapy^312^.

In a study using a patient-derived xenograft (PDX) model of non-small-cell lung cancer, the synergistic effect of physical activity and PD-1 blockade was found to impede tumorigenesis. The suppression of tumor development was characterized by a decreased rate of tumor cell proliferation and an enlargement of regions undergoing tumor necrosis^313^. These findings emphasize the potential of exercise in regulating the immune system, notably by restricting myeloid cell infiltration in the TME. Consistently, another study showed that voluntary wheel running prevented the aggregation of MDSCs in breast cancer^314^.

The proliferation of polymorphonuclear MDSCs, monocytic MDSCs, and other immunosuppressive myeloid cells has a detrimental impact on the efficacy of immunotherapy^315^-^317^. Regular physical activity may enhance the efficacy of ICIs by negating the destructive effects of these myeloid cells. Paradoxically, sedentariness and/or obesity have been associated with a more favorable response to ICIs in both preclinical and clinical studies, with individuals with a higher BMI demonstrating superior therapeutic responses^318^,^319^.

Donnelly *et al.* highlighted the requirement of a meticulous investigation into the effects of BMI on treatment outcomes. Specifically, factors such as age, sex, cancer staging, lactate dehydrogenase levels, performance status, and BRAF mutation status should be considered when examining the relationship between BMI and ICB^320^. Although preliminary findings related to the relationship between exercise and ICB therapy are encouraging, considering the aforementioned factors is crucial when investigating the supplementary role of exercise in ICB therapy. Additionally, further research is warranted to elucidate the underlying mechanisms and maximize the therapeutic potential of physical exercise when integrated with immunotherapeutic approaches (Table. 2).

#### Adoptive T cell therapy

Owing to its biological impact on T cell dynamics, physical exercise has the potential to be integrated with adoptive T cell therapy. For instance, the rapid increase in the peripheral blood T cell count after acute physical activity can be strategically used to facilitate T cell collection during leukapheresis. This approach may circumvent production barriers such as low yields from patients with lymphopenia, extended durations of T cell cultures, and suboptimal *ex vivo* T cell proliferation^321^-^323^.

Acute exercise may trigger a biphasic immune cell response, with a temporary decline in cell counts after exercise, which indicates the protective redistribution of immune cells to different organs in response to stress^219^,^324^. The functionality of these redistributed cells is often enhanced, and if collected before their re-entry into tissues, they may represent a more effective population for therapeutic production, reducing the culture duration required to reach the optimal cell count for therapeutic dosage. Notably, preliminary findings suggest a correlation between shorter culture periods and the increased antitumor efficacy of CAR-T cells^325^. Additionally, short-term physical activity can increase the recruitment of less prevalent T cell subtypes, including γδ T cells, T cells with specificity for viruses, and T cells that target specific antigens.

The success of T cell therapy relies on the proliferation and longevity of T cells *in vivo*^325^-^327^. Physical activity can enhance the proliferation and survival of T cells by stimulating the secretion of IL-7 and IL-15, which are crucial for T cell expansion and sustenance, from skeletal muscle. Therefore, physical activity may increase the effectiveness of T cell-based immunotherapies. Interleukins, specifically IL-15 and IL-7, have been identified as crucial factors contributing to the increased efficacy of CAR-T cell therapy in preclinical settings. These cytokines remarkably enhance the proliferative ability, functionality, migratory behavior, and OS of CAR-T cells, increasing their efficacy in preventing neoplastic growth^328^. In the treatment of advanced lymphoma with CAR-T cell therapy, higher serum levels of IL-15 have been associated with an increase in the number of CAR-T cells and the rate of remission^329^. However, a recent study reported a decrease in T cell counts after intense exercise in animal models, indicating the suppression of hematopoiesis^330^. Therefore, further research is warranted to evaluate the potential adverse effects of physical exercise on T cell counts in patients with cancer.

Regular physical exercise may play an instrumental role in sustaining the optimal concentrations of IL-7 and IL-15, which are crucial for the proliferation and survival of T cells after adoptive transfer^331^,^332^. In particular, post-therapeutic physical activity may enhance the mobilization and distribution of the transfused T cells throughout the body. Sustained engagement in physical activity has been shown to ameliorate the age-dependent decline in immune function, which may in turn increase the effectiveness of immunotherapeutic interventions. Physical activity enhances immune function by maintaining a high proportion of naïve T cells, reducing the accumulation of senescent T cells, and increasing the proportion of stem-cell-like memory T cells. These changes are relevant to the preparation phase of T cells in clinical studies involving adoptive cell transfer (ACT) or CAR-T cell therapy^333^.

The relationship identified between VO2max levels and the proportion of naive CD8+ T cells indicates that long-term physical activity can enhance the defense capabilities of immune cells^261^,^334^. This enhanced immune response is evidenced by the increased frequency of immune cells that are known for their high reactivity and readiness to initiate a strong immune response. The underlying mechanism may involve the exercise-induced release of specific cytokines from skeletal muscle tissues. Notably, IL-7 and IL-15 are common cytokines that are secreted after physical exertion and play a crucial role in modulating the immune response. This cytokine-mediated pathway may serve as an important factor contributing to the efficacy of physical activity in triggering effective cellular defense responses. Altogether, physical exercise holds substantial promise in immune-related clinical interventions owing to its beneficial effects on the immune system.

#### NK cell therapy

The therapeutic efficacy of NK cells in cancer exhibits variability, which can be attributed to several factors. Notably, challenges associated with cell production, including low yields of NK cells from leukapheresis samples and suboptimal *ex vivo* cell expansion or activation, significantly contribute to this variability. After infusion, the persistence of NK cells and their ability to proliferate *in vivo* are crucial determinants of treatment outcomes in patients^335^.

Acute physical activity before leukapheresis may help overcome the abovementioned challenges. Post-exercise leukapheresis samples contain approximately 5-10 times more activated NK cells than samples obtained through conventional methods. Additionally, exercise has been shown to counteract the negative effects of obesity on NK cell functionality. Obesity can impair NK cell function by disrupting glycolysis, a process mediated by the mTOR pathway, owing to the excessive accumulation of lipids and fatty acids^336^,^337^.

Glycolysis and OXPHOS play an important role in optimal NK cell activation, which is initiated by cytokines such as IL-2, IL-12, and IL-15^338^. Specifically, IL-2 and IL-15 can upregulate metabolic regulators such as mTORC1 and c-Myc, which play an essential role in sustaining the viability of NK cells and enhancing their antitumor activity^339^,^340^. Given that regular physical exercise can decrease fatty acid levels in individuals with obesity or a sedentary lifestyle, it may improve NK cell function by mitigating the immunosuppressive effects of obesity^341^,^342^.

## Discussion and Prospects

This review highlights the intricate relationship between immunosenescence, physical exercise, and anti-tumor immunity, with significant implications for aging-related diseases and cancer treatment. While the current understanding of these interactions is substantial, several key areas warrant further investigation to fully unlock the potential of exercise as a modulator of immune function and an enhancer of anti-tumor responses.

1. Mechanistic elucidation of exercise-induced immune changes: The exact mechanisms by which exercise modulates immune cell function and influences immunosenescence remain incompletely understood. Further research is needed to elucidate the signaling pathways and molecular mediators involved in exercise-induced immune changes. Specifically, the role of cytokines, such as IL-7, IL-15, and sIgA, in regulating immune cell proliferation, differentiation, and survival deserves further investigation. Additionally, the epigenetic mechanisms underlying exercise-induced changes in immune cell function and senescence need to be explored to identify potential therapeutic targets.

2. Personalized exercise interventions: The optimal exercise regimen for enhancing immune function and delaying immunosenescence remains unclear. Future studies should explore the individualized effects of different types, durations, and intensities of exercise on immune cell subsets and functional markers. This knowledge can inform the development of personalized exercise interventions tailored to the specific needs of individuals, including the elderly and cancer patients.

3. Integration of exercise with immunotherapies: The potential synergistic effects of exercise with immunotherapies, such as ICIs, adoptive T cell therapy, and NK cell therapy, warrant further investigation. Preclinical and clinical studies are needed to assess the combined efficacy and safety of exercise and immunotherapies in treating cancer. This research can also explore the optimal timing and duration of exercise interventions to maximize therapeutic outcomes.

4. Understanding the impact of comorbidities and lifestyle factors: The impact of comorbidities, such as obesity, diabetes, and cardiovascular disease, on the immune response to exercise and its potential influence on cancer development and treatment outcomes needs to be better understood. Additionally, the interplay between exercise, diet, and sleep, which are all important lifestyle factors, deserves further investigation to optimize immune function and improve health outcomes.

5. Longitudinal studies and population-based research: Longitudinal studies are necessary to track the long-term effects of exercise on immune function and its relationship with the development and progression of cancer. Population-based research can also provide valuable insights into the prevalence and correlates of immunosenescence in different populations, as well as the impact of lifestyle factors, including exercise, on immune health.

6. Development of novel therapeutic strategies: The findings from this review provide a strong foundation for the development of novel therapeutic strategies targeting immunosenescence and enhancing anti-tumor immunity. For example, drugs that mimic the effects of exercise on immune cells, such as those that increase IL-7 or IL-15 production, could be developed. Additionally, interventions that promote the clearance of senescent cells or improve the function of exhausted T cells could be explored.

There is an increasing agreement that exercise provides significant benefits for individuals following a cancer diagnosis. The 2018 Physical Activity Guidelines for Americans (PAGA) indicate that exercise positively impacts cause-specific mortality rates for prostate, breast, and colorectal cancers, as well as enhances quality of life and fitness for all cancer survivors^343^. There is biological evidence of an immune-stimulating effect in the hours after strenuous exercise. However, some other questions regarding specific exercise modalities remain open, such as the potential effects of resistance exercise on anticancer immune function^344^.

Different types of exercise have distinct effects on the immune system. Depending on the intensity, duration, and frequency of exercise, it can produce both short-term and long-term effects on immune function. Generally speaking, moderate aerobic exercise is considered beneficial for the immune system, while prolonged high-intensity exercise may lead to temporary suppression of immune function. Moderate aerobic exercises, such as walking, jogging, swimming, or cycling, are commonly regarded as enhancing the immune system's defense capabilities. Research shows that regular moderate-intensity exercise can boost the activity of immune cells, including natural killer (NK) cells, macrophages, and lymphocytes, which play key roles in fighting pathogens and tumor cells^345^. Studies indicate that after prolonged high-intensity exercise, NK cell numbers decrease, T cell function is temporarily suppressed, and pro-inflammatory cytokines like IL-6 increase, exacerbating inflammation. This immune suppression response is thought to be a protective mechanism for the body to repair physiological stress and damage caused by exercise^346^. Strength training and resistance training have more complex effects on the immune system. On one hand, moderate strength training can enhance immune function, similar to aerobic exercise, by promoting immune cell activity. On the other hand, excessive strength training, especially without adequate recovery, may lead to immune suppression and an increased risk of infections^331^. Therefore, maintaining a balanced exercise routine, with proper intensity and recovery time, can optimize the positive effects of exercise on the immune system and support overall health.

In conclusion, the relationship between immunosenescence, physical exercise, and anti-tumor immunity is complex and multifaceted. Further research is needed to fully understand these interactions and develop evidence-based strategies to optimize immune function, delay immunosenescence, and improve the outcomes of cancer treatment.

## Figures and Tables

**Table 1:** Immunosenescence and immunotherapy.

Characterization of immunosenescence	Immunotherapy methods	Results	Mechanisms	Reference
IL-6 and N/L ratio	sequential administration of nivolumab followed by ipilimumab or the reverse sequence	negatively associated with OS	inhibits intertumoral T cell activation	290
high circulating level of IL8	anti-PD-L1 checkpoint inhibitors	negatively associated with OS	the recruitment of neutrophils and myeloid cells to enhance immune evasion mechanisms involving neutrophil extracellular traps	292
higher pre-treatment prevalence of CD27-CD28-Tim-3+ CD57+ T-cells	pembrolizumab or in combination with a lower dose of ipilimumab	reduced overall survival	the loss of CD27 and CD28 as well as the expression of the Tim-3 and CD57, correlated with resistance to ICI in the pilot study	288
upregulate SASP-related genes	anti-PD-L1 checkpoint inhibitors	improved responses to ICI *in vivo*	reverses the resistant cell state, induces components of SASP	295
reduced components of SASP	anti-PD-L1 checkpoint inhibitors	no clinical benefit achieved	associated with T cell exclusion and immune evasion	300
overexpression of different types of SASP	anti-PD-L1 and other ICIs	associated with poorer OS	alterations of SASP could impact TIME establishment, which ultimately contributes to immune escape and provokes tumor development	296
high IL-6 levels	ipilimumab	establish a tumor microenvironment that can shelter incipient tumor cells	SASP-factor IL-6 establishes myeloid-driven immunosuppression where CD8^+^ T cells were inhibited, resulting in unrestrained tumor growth	287
KLRG1+CD57+ expression	CAR-T cell therapy	negatively associated with OS	CD57 is the marker of T-cell senescence that has been shown to be an indicator of limited T-cell proliferative capacity	305
not applicable	CAR-T cells +histone deacetylase inhibitors	significant suppression of Her2+ pancreatic cancer	the employment of histone deacetylase inhibitors to catalyze a phenotypic transition in endogenous T cells towards a more durable memory T cell phenotype	308
age	NK cell therapies	purified NK cells are feasible in elderly patients with high-risk acute myeloid leukemia	/	75

**Table 2:** Clinical trials of relationship between exercise and the efficacy of immunotherapy

NCT number	Cancer types	Interventions	Outcome Measures	Experimental design
NCT04127318	renal cell carcinoma, cutaneous malignancies or bladder cancer	under-the desk bike + immunotherapy infusions.	evaluating the effects of low-moderate intensity pedaling during immunotherapy	During their 30-minute immunotherapy infusions, participants will pedal using a stationary cycle ergometer. Participants will be allowed to determine their pedaling intensity and cadence, however, will be encouraged to reach the established goal intensity level. A research personnel will monitor the patient's heart rate, blood pressure, and RPE at baseline and every 10 minutes throughout the pedaling session. Participants will also have treatment response biomarkers gathered at baseline and before and within 10 minutes of completing their first and fourth immunotherapy infusions. Lastly, participants will complete both a physical activity questionnaire and a quality-of-life questionnaire at baseline and following their fourth treatment.
NCT05358938	cutaneous melanoma, cutaneous squamous cell carcinoma, merkel cell carcinoma	30 minutes of moderate exercise on an arm ergometer, a cycle ergometer, or a treadmill + checkpoint blockade immunotherapy	pathological complete response	Adjuvant participants will receive 1 year [currently 9-18 cycles] of therapy with avelumab, cemiplimab, ipilimumab, nivolumab, pembrolizumab or relatlimab, or currently approved standard of care treatment, either alone or in combination. Participants also will complete 30 minutes of moderate exercise on an arm ergometer, a cycle ergometer, or a treadmill prior to each administration of standard of care checkpoint blockade immunotherapy across all cycles. Blood samples will be collected at 1) baseline (upon arrival to clinic), 2) post-exercise, and 3) post-infusion. Blood samples will be obtained on the first, third, midpoint, and final infusion dates.
NCT06008977	cutaneous melanoma cutaneous squamous cell carcinoma merkel cell carcinoma	exercise intervention trial +checkpoint blockade immunotherapy	preliminary data on the impact of a day-of-therapy exercise intervention	Patients randomized to the standard arm will receive clinical care following AH standards for the patient's disease type and therapeutic setting. Patients randomized to the exercise arm will complete up to 30 minutes of same-day exercise prior to each administration of checkpoint blockade immunotherapy across all cycles. The preferred exercise is 30 minutes of moderate exertion on a cycle ergometer.
NCT04676009	metastatic lung cancer	exercise arm +immunotherapy and chemotherapy infusion	investigate the feasibility of an acute physical exercise during the immunotherapy/chemotherapy preinfusion	Patient randomized to the Exercise arm will receive physical activity recommendations at inclusion and nutritional assessment will be carried out during the first and last treatment cure. Patients will receive an acute physical exercise just before immunotherapy and chemotherapy infusion. They will have a home walking program and will have to wear an activity tracker during the 3 months of intervention.3 blood sampling times will be specially added to the study and will take place before exercise (1), after exercise (2) and 12 hours after the start of treatment (3).
NCT06152926	urological cancers	physical activity + immunotherapy	to understand participant's perspectives and experiences of physical activity and exercise on immune check point immunotherapy treatment through inductive thematic analysis of semi-structured individual interviews.	People diagnosed with a urological cancer receiving Immune checkpoint inhibitor treatment at a single centre trust will be invited to either face to face or online video-conference individual interviews (Microsoft teams) according to participant preference. Consenting participants will be interviewed via semi-structured interview methods round a topic guide and audio-recorded. Inductive thematic analysis will be conducted to explore participant experiences and perceptions of physical activity and exercise.
NCT06298734	melanoma (skin) skin cancer advanced melanoma	high-intensity exercise and high-fiber diet+ immunotherapy	change in gut microbiome diversity	Experimental: Group A: high-intensity exercise.10 participants will complete: in-office baseline visit, virtual exercise sessions 3x weekly, post-intervention in-office visit. Group B: high-fiber diet.10 participants will complete: in-office baseline visit, 1x weekly appointment with research staff to review to review diet adherence, post-intervention in-office visit. Group C: combined high-intensity exercise and high-fiber diet.10 participants will complete: in-office baseline visit, virtual exercise sessions 3x weekly. 1x weekly appointment with research staff via zoom platform to review to review diet adherence. Diet appointment may be combined with exercise session appointment, post-intervention in-office visit. No Intervention: Group D: attention control 10 participants will complete: in-office baseline visit, participants will receive a general healthy lifestyle guidebook, pot-intervention in-office visit.
NCT04866810	melanoma	diet + exercise + immunotherapy	feasibility of conducting a decentralized clinical trial involving diet and exercise prescriptions with stool sample collections in patients receiving immunotherapy	High fiber, plant-based diet + exercise prescription with ACT sessions.
NCT03525873	advanced malignant neoplasm, metastatic malignant neoplasm, recurrent malignant neoplasm	methylphenidate + physical activity +anti-PD-1 immunotherapy	assessment of the effects of methylphenidate plus physical activity in reducing cancer-related fatigue	Patients receive methylphenidate PO BID for up to 2 weeks in the absence of disease progression or unacceptable toxicity. Patients also complete physical activity consisting of walking and resistance exercise over 25-40 minutes QD 4 days a week. After 2 weeks, patients may continue methylphenidate at the discretion of the treating physician for up to 12 weeks in the absence of disease progression or unacceptable toxicity.
NCT06026111	lung cancer	12-week exercise training +immune checkpoint inhibitors	proportion of participants completing the exercise intervention sessions	Patients in the high-intensity interval training (HIIT) group will perform alternating vigorous-intensity and recovery aerobic exercise intervals on a provided home stationary bike. The HIIT protocol consists of alternating a high-intensity exercise phase (1 min at 65-90% of workload corresponding to VO2peak) and a recovery phase (1 min at 30%), and the high-intensity and recovery intervals will be repeated 5-10 times in each session. The moderate-intensity continuous training (MICT) group will perform an aerobic exercise at a continuous intensity in each session on a stationary bike. Similar to HIIT, the intensity will be progressed (47.5-60%). The total work of MICT is equalized with HIIT for training volume and frequency to compare the differences exerted from different intensities and energy expenditure.
NCT05763563	Lymphoma, leukemia, myeloma	aerobic exercise +CAR-T therapy	participants health-related quality of life - baseline/participant self-reported exercise	Participants will take part in an exercise program in which they will be encouraged to perform approximately 30 minutes of resistance training exercises approximately twice per week until they undergo CAR-T therapy (Approximately 4-6 weeks). Participants will also be encouraged to perform moderate aerobic exercise such as brisk walking or using stationary aerobic equipment at least 3 times per week. Participants will wear a FitBit fitness watch to monitor aerobic exercise.
NCT05318807	lung cancer	chemotherapy, radiotherapy and/or immunotherapy treatment + exercise	evaluation of participant acceptability of the modified godin leisure time exercise questionnaire used for the personalised prehabilitation programme	A personalised plan of diet, exercise and emotional support for patients having chemotherapy, radiotherapy and/or immunotherapy treatment for lung cancer.
NCT03007602	hematologic malignance	upper extremity aerobic exercise training+ immunotherapy	functional exercise capacity evaluation	The upper extremity aerobic exercise training with an arm ergometer will be performed in the treatment group so that training intensity will be between 60% and 80% of the maximum heart rate, dyspnea perception will be 3-4 according to Modified Borg Scale and fatigue perception will be 5-6 according to Modified Borg Scale, training duration will be a 6-week.
NCT03576274	non-metastatic solid tumor cancer	a 12 weeks technology enhanced home exercise	fatigue, skeletal muscle strength, heat shock protein level, brain derived neurotrophic factors	Technology Enhanced Home Exercise (TEHE): a 12 weeks program including one 60-minute goal setting and exercise training program and follow up phone calls. Technology Enhanced Home Exercise plus: a 12 weeks program including one 60-minute goal setting and exercise training program and follow up phone calls. Auricular Point Acupressure (APA) A noninvasive complementary method to provide pressure on the ear points.
NCT05093192	chronic lymphocytic leukaemia	exercise+ chemo-immunotherapy	investigate whether an acute bout of exercise changes the frequency of immune cells	Participants will perform a supervised acute bout of aerobic exercise over 20-30 minutes, corresponding to a target power output at an intensity +10 to 15% above ventilatory threshold.

## Abbreviations

AID: activation-induced cytidine deaminase
AML: acute myeloid leukemia
APCs: antigen-presenting cells
APRIL: a proliferation-inducing ligand
BAFF: B-cell-activating factor
BCRs: B-cell receptors
BMI: body mass index
cAMP: cyclic adenosine monophosphate
CAR: chimeric antigen receptor
cDCs: classic DCs
DCs: dendritic cells
DLBCL: diffuse large B-cell lymphoma
EM: effector memory
GLUTs: glucose transporters
GZMB: granzyme B
HLA: human leukocyte antigen
ICIs: Immune checkpoint inhibitors
IFN-γ: interferon gamma
Igs: immunoglobulins
IL: interleukin
KIR: killer cell immunoglobulin-like receptor
LPSs: lipopolysaccharides
mDCs: myeloid DCs
MDSCs: myeloid-derived suppressor cells
MHC-II: major histocompatibility complex class II
mtDNA: mitochondrial DNA
mTOR: mammalian target of rapamycin
mTORC1: mTOR complex 1
NETs: neutrophil extracellular traps
NK: natural killer
OS: overall survival
OXPHOS: oxidative phosphorylation
PBMCs: peripheral blood mononuclear cells
pDCs: plasmacytoid DCs
PDX: patient-derived xenograft
PFS: progression-free survival
PRRs: pattern recognition receptors
SAHF: senescence-associated heterochromatic foci
SASP: senescence-associated secretory phenotype
SA-β-gal: senescence-associated β-galactosidase
SIgA: secretory immunoglobulin A
SIP: systemic immune-inflammation index
TAM: tumor-associated macrophage
TCRs: T cell receptors
TGF-β: transforming growth factor beta
TLRs: Toll-like receptors
TME: tumor microenvironment
TNF-α: tumor necrosis factor-alpha
Tregs: regulatory T cells
URTIs: upper respiratory tract infections
VO2max: maximal oxygen consumption rate
